# Supplementary material for: Estimated impact of tafenoquine for Plasmodium vivax control and elimination in Brazil: A modelling study
Source: PLoS Med. 2021 Apr 23;18(4):e1003535. doi: 10.1371/journal.pmed.1003535 (PMC8064585; doi:10.1371/journal.pmed.1003535)
Supplement: S1 Text — (PDF) [file pmed.1003535.s001.pdf]

Supplementary Information

Estimated impact of tafenoquine for *Plasmodium vivax* control and elimination in Brazil: A modelling study

Narimane Nekkab[1], Raquel Lana[2], Marcus Lacerda[5,6], Thomas Obadia[1], André Siqueira[7], Wuelton Monteiro[5,8], Daniel Villela[2], Ivo Mueller[1,3,4], Michael White[1]

Affiliations

[1] Malaria: Parasites and Hosts, Department of Parasites and Insect Vectors, Institut Pasteur, Paris, France

[2] Programa de Computação Científica, Fundação Oswaldo Cruz (Fiocruz), Rio de Janeiro, Brazil

[3] Population Health & Immunity Division, Walter and Eliza Hall Institute of Medical Research, Parkville, Australia

[4] Department of Medical Biology, University of Melbourne, Melbourne, Australia

[5] Diretoria de Ensino e Pesquisa, Fundação de Medicina Tropical Dr. Heitor Vieira Dourado, Manaus, Brazil

[6] Instituto Leônidas e Maria Deane, Fundação Oswaldo Cruz (Fiocruz Amazônia), Manaus, AM, Brazil.

[7] Instituto Nacional de Infectologia Evandro Chagas, Fundação Oswaldo Cruz (Fiocruz), Rio de Janeiro, Brazil

[8] School of Health Sciences, Universidade do Estado do Amazonas, Manaus, AM, Brazil

Correspondence

narimane.nekkab@pasteur.fr

michael.white@pasteur.fr

Contents

1 Individual-based model 3
2 Model adaptation for Brazilian epidemiology 4
2.1 Existing case management guidelines with primaquine 4
2.2 Introduction of tafenoquine for case management 5
2.3 G6PD diagnostics 5
2.4 Gaussian mixture model fitting 7
3 Parameter estimates 10
3.1 Demography 11
3.2 Relapses 12
3.3 Entomological parameters 13
3.4 Primaquine adherence 13
3.5 Low CYP2D6 metaboliser prevalence 13
3.6 Impact of CYP2D6 activity score and *P. vivax* malaria recurrence 14
3.7 G6PD deficiency prevalence in Brazil 15
3.8 Chloroquine resistance and efficacy in clearing blood-stage parasites 16
3.9 Primaquine efficacy 16
3.10 Tafenoquine efficacy 17
3.11 Weekly primaquine 17
4 Extended results 17
4.1 Radical cure estimates 17
4.2 Asymptomatic infections 20
4.3 Cases averted, treatment courses and G6PD tests in archetype settings 21
4.4 Cases averted at the national level 23
4.5 Primaquine adherence 24
4.6 Effect size over time for S1 25
4.7 Effect size summary table for all scenarios 1-10 27
4.8 Varying assumption of 8-aminoquinoline efficacy 27
4.9 Varying assumption on low CYP2D6 metabolisation 30
4.10 Variation in blood-stage prophylactic duration 32
5 References 33
6 Appendix 38

1 Individual-based model

Previously, we developed a mathematical model of the transmission dynamics of *P. vivax* in Papua New Guinea (PNG), and the potential impact of varying levels of vector control and case management to treat blood-stage infections. Fig A shows a compartmental representation of the transmission model. The model is implemented in two complementary formats: firstly, as a deterministic compartmental model described by a system of ordinary differential equations, and secondly, as an individual-based stochastic model. Notably, the two model implementations provide identical predictions for large population sizes when stochastic effects average out. The population dynamics of multiple mosquito species are described using compartmental models. Further details can be found in White *et al*. [1].

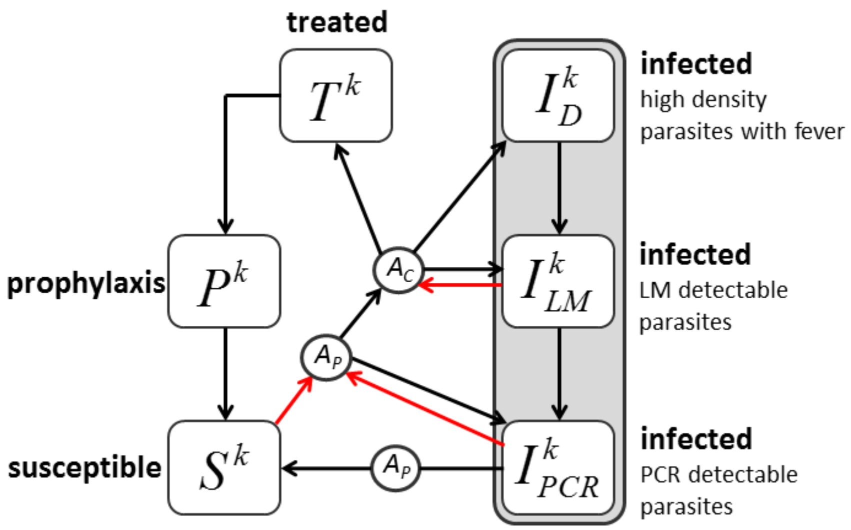

Fig A: Compartmental representation of *P. vivax* transmission model in humans. Infected individuals can be in one of three compartments depending on whether blood-stage parasitaemia is detectable by PCR (I_{PCR}), light microscopy (I_{LM}) or has high density with accompanying fever (I_D). A proportion of individuals that progress to a symptomatic episode of *P. vivax* will undergo treatment with a blood-stage drug (*T*) leading to clearance of blood-stage parasitaemia and a period of prophylactic protection (*P*) before returning to the susceptible state (*S*). The superscript *k* denotes the number of batches of relapse causing hypnozoites in the liver. Red arrows denote new blood-stage infections arising from either new mosquito bites or relapses. Each square denotes a compartment and the circles denote the dependence of transition rates between compartments on levels of anti-parasite immunity (A_p) and levels of clinical immunity (A_c).

The model was calibrated to data from epidemiological studies involving almost 60,000 individuals in PNG. The model was further validated to data from systematic reviews of the associations between multiple metrics of malaria transmission [2, 3]: *P. vivax* prevalence by light microscopy (*Pv*PR_{LM}); *P. vivax* prevalence by PCR (*Pv*PR_{PCR}); the detected incidence of clinical malaria per 1000 people per year; and the entomological inoculation rate (EIR). The model corresponded well to data from these systematic reviews of *P. vivax* from across the world, providing evidence that a model developed in a Papua New Guinean context, can be adapted to describe the epidemiology of *P. vivax* in other endemic regions such as Brazil. However, there are some important differences between the epidemiology of malaria in PNG and Brazil that need to be explicitly accounted for.

2 Model adaptation for Brazilian epidemiology

Malaria transmission epidemiology is extraordinarily complex, with exposure occurring in a wide range of occupational and environmental settings. Despite this complexity, malaria transmission epidemiology can be broadly categorised in two ways as follows:

(i) Peri-domestic transmission with most exposure occurring in the household and close to where people live. This type of exposure pre-dominates in much of Africa and PNG.
(ii) Occupational exposure where people's work activities bring them into contact with mosquitoes in areas that may be quite far from where they live and sleep.

The relative contribution of these two modes of transmission can be assessed by examining the age and gender distribution of clinical cases of malaria. In an example of peri-domestic transmission, cases are distributed across all ages and between males and females. Notably, a large proportion of cases occur in young children. Settings with occupational exposure are typically characterised by having substantially more cases in men than in women, and more cases in working age adults (16 to 65 years of age).

The individual-based transmission model was adapted to account for these two distinct modes of transmission via the following changes:

(i) Stratification into males and females.
(ii) Males are further stratified according to whether they are exposed to malaria via their occupation, or are exposed to malaria solely through domestic transmission.
(iii) Two separate populations of mosquitoes corresponding to peri-domestic and occupational exposure settings are simulated.

In particular we assume that 37% of men aged 16 to 65 years are at risk of occupational exposure [4]. The additional risk associated with occupational exposure is estimated based on the proportion of reported cases in adult males in a municipality, as recorded in the SIVEP database [5].

2.1 Existing case management guidelines with primaquine

In order to model the potential impact of introducing tafenoquine with testing for G6PD deficiency into cases management for *P. vivax* in Brazil, it is important to first assess the existing case management strategies and our model assumptions.

In Brazil, individuals with symptomatic cases of malaria are provided with free treatment at their local public health centre. Upon microscopic confirmation of *P. vivax* infection, patients enter the primaquine treatment pathway shown in Fig 1B. Primaquine is not prescribed to infants < 6 months of age. Primaquine is not prescribed to pregnant or lactating women because of the potential risk to their foetus or young infant. We assume all *P. vivax* positive cases receive chloroquine without any counter-indications. G6PD testing is not required for primaquine eligibility. Current primaquine treatment thus covers a very high proportion of clinical cases. It is assumed that primaquine is not efficacious in individuals who do not adhere to the full 7-day regimen and do not account for any dose-response. Primaquine is not assumed to be efficacious in individuals with a low CYP2D6 metaboliser phenotype. Furthermore, even in patients capable of properly metabolising primaquine, we assume it will not always be efficacious.

The Brazilian endemic states have an average G6PDd prevalence of 5.5% (from municipality-specific estimates see Section 3.6). If we consider G6PDd cases prescribed primaquine (due to lack of testing) who did not fully adherence to the radical cure regimen due to adverse events, we estimated that on average 40.5% [95% CI 40.3%-41.0%] of cases were effectively treated. Of all *P. vivax* cases treated annually in Brazil, we estimate that 3.2% or 7000 cases may be at risk of developing haemolysis due to G6PDd.

2.2 Introduction of tafenoquine for case management

In order to be included for *P. vivax* case management, tafenoquine must be accompanied by point of care testing for G6PD deficiency. Patients with microscopically confirmed *P. vivax* infection are first stratified by age. Only individuals older than 16 years of age are eligible to receive tafenoquine upon initial roll-out. Pregnant or lactating women are excluded from receiving either primaquine or tafenoquine. All patients who are eligible for receiving either primaquine or tafenoquine will have their G6PD activity measured using a SD Biosensor test. Note that this even includes children <16 years of age who are not eligible for tafenoquine. Depending on their age, pregnancy/lactating status and measured G6PD activity, patients will be prescribed one of

(i) chloroquine (25 mg/kg total dose over 3 days);
(ii) chloroquine plus primaquine (3.5 mg/kg total dose over 7 days);
(iii) chloroquine plus tafenoquine (single dose 300 mg).

Notably, tafenoquine will not completely replace primaquine, as there will be many patients not eligible for tafenoquine who will still benefit from primaquine treatment. Fig 1D shows a schematic representation of the new tafenoquine treatment pathway. The proportion of those receiving chloroquine only is expected to increase in part due to the identification of G6PD deficient individuals but also in part due to the misclassification of intermediates (30-70%) by the G6PD quantitative test. Some participants prescribed primaquine will not adhere to the full regimen. It is assumed that low CYP2D6 metabolisation does not affect tafenoquine efficacy in the standard tafenoquine introduction scenarios; however, we assess the differential impact of low CYP2D6 metabolisation on tafenoquine efficacy in Section 4.5.

2.3 G6PD diagnostics

Currently in Brazil, G6PD testing is not a requirement for individuals to receive primaquine. Once tafenoquine is introduced, we assume that quantitative G6PD testing will be implemented in all patients eligible for primaquine and tafenoquine (over 6 months old and not pregnant). An important component that should also be considered is the sensitivity and specificity of the SD Biosensor STANDARD G6PD test measuring quantitative concentration of total hemoglobin (g/dL) and G6PD enzymatic activity (U/g Hb) to be deployed in Brazil. The cut-offs for tafenoquine eligibility will requires at least 70% G6PD activity (normal G6PD activity), 30-70% (intermediate G6PD activity) for primaquine, and < 30% (deficient G6PD activity) for only chloroquine [6-8].

Pal *et al*. assessed the sensitivity and specificity of the SD Biosensor G6PD test on fresh venous blood and found a 100% sensitivity and 97% specificity in those with < 30% G6PD activity, and 95.5% sensitivity and 97% specificity in those with < 70% G6PD activity [9]. Using their data on US venous blood samples, we estimated the probability of classifying individuals in the G6PD activity groups in the model by calculating the proportion of correctly classified and misclassified G6PD activity scores (Fig B). Since African Americans were included in the study and similar G6PDd genetic variants are observed between African Americans and Brazilians [10] (see section

2.4), we assume that the SD Biosensor G6PD test will have comparable classification between the two populations.

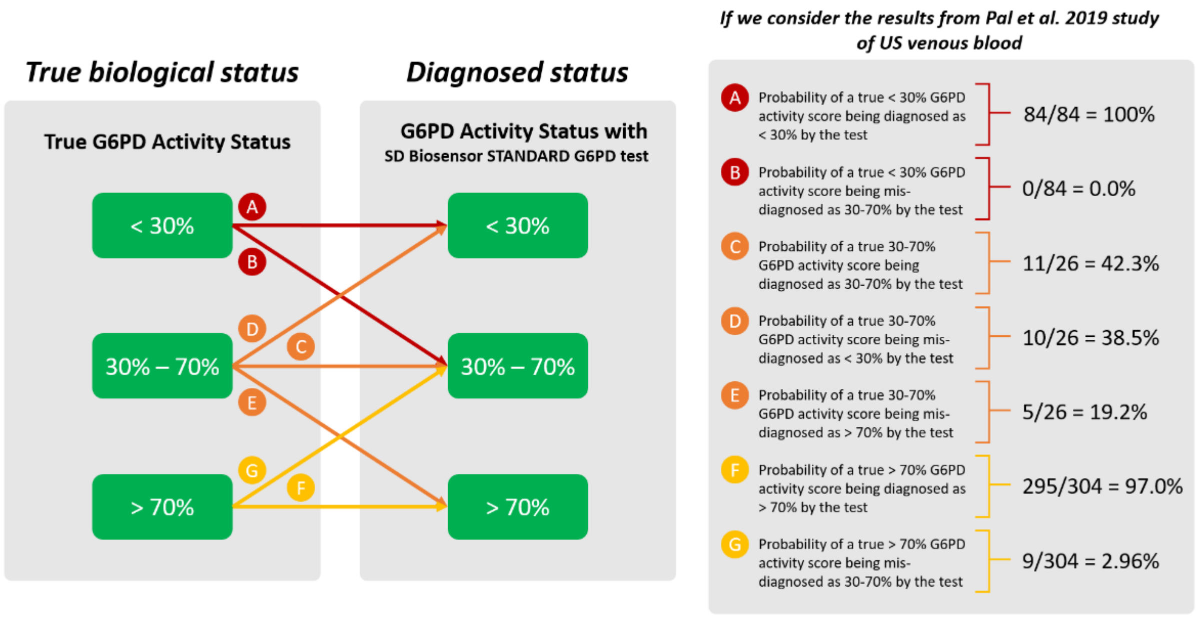

Fig B: G6PD testing model. The quantitative point of care SD Biosensor G6PD test has imperfect accuracy when compared to a laboratory-based reference assay. Data from a study by Pal *et al.* is used to estimate the accuracy of the SD Biosensor test at classifying individuals according to the following activity categories: < 30%; 30% - 70%; and > 70% [11].

The relationship between genotypic prevalence of G6PD deficiency (as measured by allele frequency in males) and quantitative G6PD activity measured either by a reference assay or the point of care SD Biosensor is shown in Fig C.

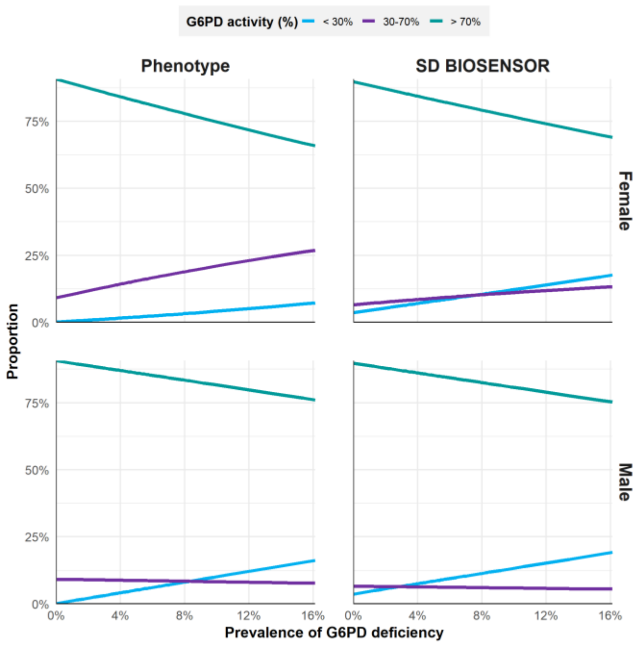

Fig C: Association between G6PD genotype and phenotype. The G6PD activity model described in Section 2.4 allows generation of estimates of G6PD activity given genotypic prevalence of G6PD deficiency (measured by allele frequency in males). This model provides estimates of the proportions in three G6PD activity categories (< 30%; 30% - 70%; and > 70%) as measured by a reference assay. When tested by a SD Biosensor with imperfect accuracy, these estimates can be further adjusted as shown.

2.4 Gaussian mixture model fitting

There is a complex relationship between G6PD phenotype as measured by G6PD activity, and G6PD genotype, especially since the gene for G6PD deficiency is x-linked. We have developed a method to estimate the distribution of G6PD activity scores in the population from a large G6PD deficient prevalence survey (Table D, Fig F) for each municipality.

We define q to be the prevalence of G6PD deficiency, based on the proportion of males with a deficient genotype. Note that the x-linkage of the gene for G6PD deficiency means that males are either hemizygous deficient or hemizygous normal. Based on the Hardy-Weinberg principles of genotype frequencies in the population, we can estimate the following values: deficient males q, normal males $(1 - q)$, homozygous deficient females q^2, heterozygous deficient females $2q(1 - q)$, and homozygous normal females $(1 - q)^2$

Based on this principle, we developed Gaussian mixture models to estimate the distribution of G6PD activity scores for males and females. We assumed a normal distribution of activity scores for each genotype. The Gaussian mixture model for G6PD activity scores in males is a combination of a normal distribution of G6PD deficients q and a normal distribution of G6PD normals $(1 - q)$. For females, the model combines a normal distribution of homozygous deficient females q^2, a normal distribution of heterozygous deficient females $2q(1 - q)$, and a normal distribution of homozygous normal females $(1 - q)^2$. Each normal distribution is determined by a mean μ and standard deviation σ. As a result, the following seven model parameters were estimated: q, μ_{def}, σ_{def}, μ_{het}, σ_{het}, μ_{nor}, σ_{nor}.

Under a Bayesian framework, the model parameters were estimated using Markov Chain Monte Carlo (MCMC) methods simultaneously for men and women and for different populations. We assumed uniform prior distributions within the following range: $\boldsymbol{q}$ [0, 1], $\boldsymbol{\mu}_{def}$ [0, 2], $\boldsymbol{\sigma}_{def}$ [0, 2], $\boldsymbol{\mu}_{het}$ [3, 7], $\boldsymbol{\sigma}_{het}$ [0, 5], $\boldsymbol{\mu}_{nor}$ [8, 15], and $\boldsymbol{\sigma}_{nor}$ [0, 5]. The model provides estimates of the prevalence of G6PD deficiency $\boldsymbol{q}$ that vary by population.

We collated datasets of G6PD activity scores in both men and women from publicly available data (Table A). For model fitting, four datasets of healthy individuals in Bangladesh, Cambodia, Indonesia, and Israel were standardized so that the mean activity scores of G6PD normal males was set to 10. In order to determine which males were considered G6PD "normal" we either used a visual inspection of the distribution of the data or used the cutoff proposed by the original publication.

We estimated the following posterior median parameter estimates from the MCMC chains: $\boldsymbol{\mu}_{def}$ = 0.48, $\boldsymbol{\sigma}_{def}$ = 0.31, $\boldsymbol{\mu}_{het}$ = 4.60, $\boldsymbol{\sigma}_{het}$ = 1.67, $\boldsymbol{\mu}_{nor}$ = 10.16, $\boldsymbol{\sigma}_{nor}$ = 2.39. In the mathematical model, we fix the Gaussian mixture model parameter estimates to these values. Based on the municipality G6PD deficiency prevalence, we estimate the distribution of activity scores in men and women. Model estimates for the four data sets are shown in Fig D.

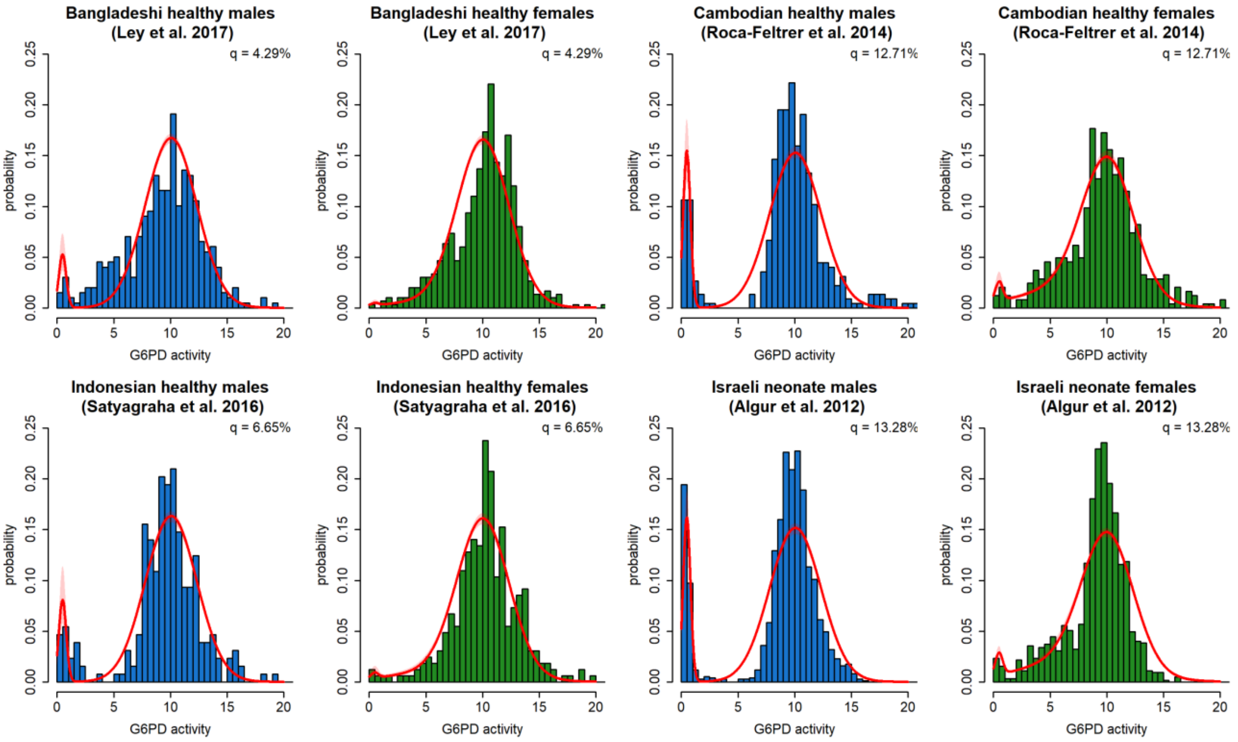

Fig D: G6PD activity and MCMC model fit and validation for four datasets. We compare the distribution of the datasets used to estimate parameters for the G6PD activity model described in Section 2.4 and the model prediction for men and women for each study. G6PDd prevalence model estimates are consistent with those reported by each study (Table L).

While the G6PDd prevalence model estimates are consistent with those reported by each study, G6PD mutations in these populations are considered more severe than the prevalent G6PD A(−) variant (202 G>A mutations) commonly found in South America [12-14]. The common variant forms in the datasets: Bangladesh (Orissa, Kalyan-Kerala and Mahidol); Cambodia

(Viangchan); Indonesia (Vanua Lava); Israel (Mediterranean) tend to be more severe and as a result, the fitted model may overestimate the G6PD activity in the Brazilian population [15, 16]. In particular, the prediction of heterozygous women under the 70% threshold in Brazil may be overestimated in comparison to the data used to fit the model. As a result, more women could in fact be eligible for tafenoquine treatment than predicted by the model. Overall, since this concerns a small proportion of the overall population, this will not have a major impact on our prediction of tafenoquine impact on transmission; however, further optimization of the model will be required using Brazilian data when it becomes available.

3 Parameter estimates

A systematic literature review was conducted to identify appropriate model parameters estimates for the Brazilian setting. In particular, we identified population factors, treatment regimen and efficacy, G6PD diagnostic performance, and data on low CYP2D6 relevant for Brazil. The summary of the review is shown in Table A.

Table A: Parameter values for primaquine and tafenoquine treatment pathways. Where multiple parameter values are provided, the assumed estimate is indicated by *.

	notes	parameter value	reference
Population factors			
proportion of symptomatic cases treated		95%	assumption
age of cases			SIVEP-Malaria 2018 data [5]
gender of cases			SIVEP-Malaria 2018 data [5]
pregnancy status of cases			SIVEP-Malaria 2018 data [5]
lactating status of cases		6 months	frequency based on SIVEP-Malaria 2018 pregnancy data [5]
Chloroquine			
dosing regimen	blood-stage treatment	25 mg/kg total dose over 3 days	
lower age limit	no lower limit		
treatment of pregnant women	yes		
treatment of lactating women	yes		
duration of prophylaxis	against blood-stages	28 days	
efficacy	mono-therapy	89.9%	[17]
	co-administered with primaquine	94.8%	[18]
	co-administered with tafenoquine	100%	assumption
Primaquine			
dosing regimen	liver-stage treatment	3.5 mg/kg total dose over 7 days	
lower age limit		6 months	
treatment of pregnant women	no		
treatment of lactating women	no		
adherence	Brazil (full 7-day regimen)*	66.7%	[19]
	Brazil (full 7-day regimen)	86.4%	[20]
	Peru data (full 7 day regimen)	62.2%	[21]
efficacy		71.3%	[22]

duration of prophylaxis	against blood-stages and liver-stages	8 days	
efficacy in low CYP2D6 metabolisers	no		[23]
effectiveness	all-or-nothing: 0.667*0.713*(1 – 0.081)	43.7%	
Tafenoquine			
dosing regimen	liver-stage treatment	300 mg single dose	
lower age limit		16 years	
treatment of pregnant women	no		
treatment of lactating women	no		
adherence	1 dose DOT	100%	
efficacy	based on equivalence between PQ and TQ in phase 3 trial	71.3%	[22, 24]
duration of prophylaxis	against blood-stages and liver-stages	45 days	[24]
efficacy in low CYP2D6 metabolisers	yes		[25]
effectiveness	all-or-nothing	71.3%	
G6PD deficiency			
G6PD deficiency prevalence	allele frequency in males (with state-level estimates)	5.52%	Table D, Fig F
SD Biosensor G6PD test sensitivity	< 30% threshold<br>< 70% threshold<br>< 80% threshold	100%<br>95.5%<br>95.0%	[11]
SD Biosensor G6PD test specificity	< 30% threshold<br>< 70% threshold<br>< 80% threshold	97%<br>97%<br>86.3%	[11]
CYP2D6 metabolisation			
low CYP2D6 prevalence (AS < 1)		8.1%	[26]
low CYP2D6 prevalence (AS ≤ 1)		19.5%	[27]

3.1 Demography

Demographic data was obtained from the Brazilian census collated by Instituto Brasileiro de Geografia e Estatística (IBGE). An overview of the Brazilian population is shown in Fig E. The last census was undertaken in 2010, providing age and gender stratified population estimates for every municipality, plus data on a large number of other covariates. Municipal population size and average age was extracted from IGBE reported 2018 estimates.

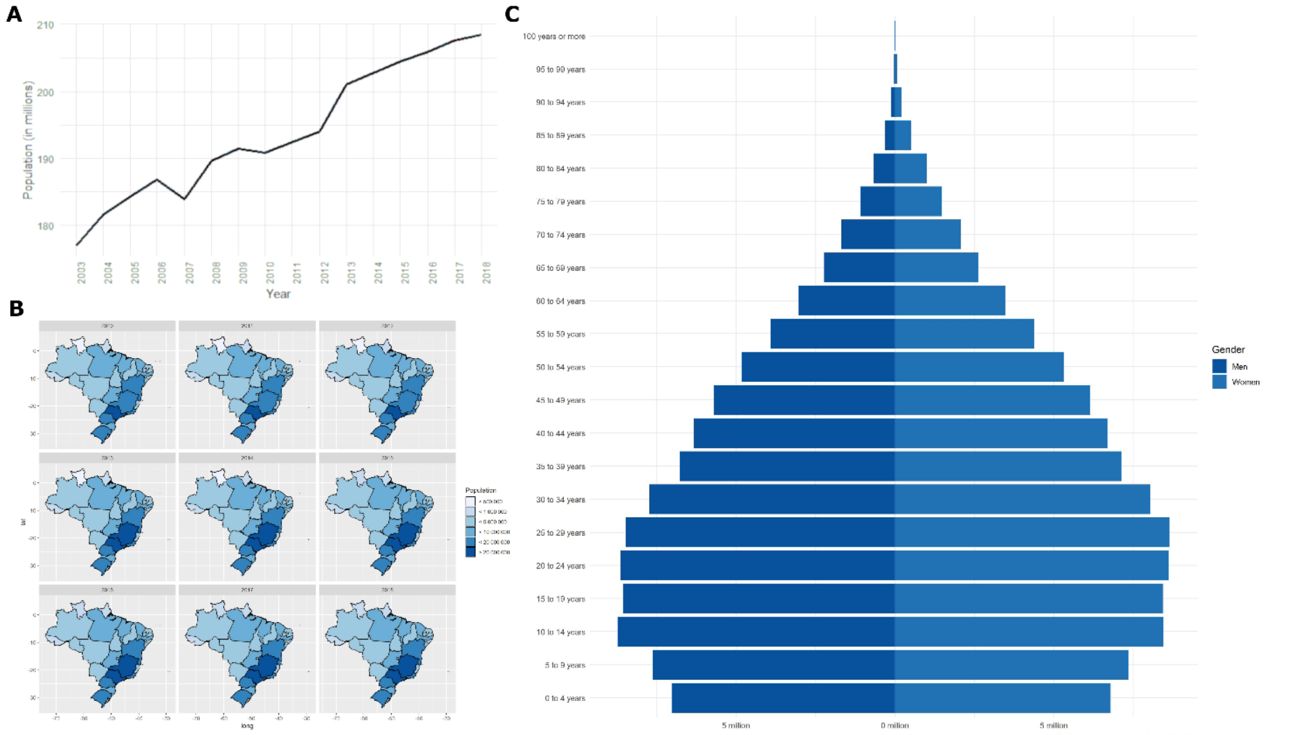

Fig E: Brazilian demographic data. Data from the published by Instituto Brasileiro de Geografia e Estatística (IBGE) from 2003 to 2018 with population estimates, count data for 2007 and census data for 2010 **(A)** by state **(B)**. The population pyramid from the 2010 census **(C)**. Maps generated with shapes files from the R package malariaAtlas.

3.2 Relapses

Brazil is typically characterised as having the tropical relapse phenotype of *P. vivax* (Battle 2014; Lover 2013) [28, 29]. However, it is challenging to accurately study the epidemiology of *P. vivax* relapses in Brazil, because radical cure with primaquine is routinely administered as part of case management. As such, many epidemiological studies reporting recurrent *P. vivax* episodes may not capture natural relapses patterns, but relapse patterns following some degree of primaquine treatment. Despite the challenges in identifying which recurrences are relapses, and the potential for partially efficacious primaquine treatment to prevent some relapses, there are very good sources of data on recurrences from the SIVEP databases. Based on analysis of data from 26,295 recurrences Daher *et al.* estimated a median time between recurrences of 69 days [30]. The epidemiology of *P. vivax* relapses can be described via three parameters as shown in Table B [31].

Table B: Parameters describing the epidemiology of *P. vivax* relapses in Brazil.

description	parameter	value	reference
relapse rate<br>(time to next relapse)	f	1/69 day^{-1}	[30, 32]
liver-stage clearance rate<br>(time to clearance of hypnozoites)	γ_L	1/383 day^{-1}	[31]
relapses per primary infection	$h = f / \gamma_L$	5.6	

3.3 Entomological parameters

There are a number of species of *Anopheles* mosquito that contribute to malaria transmission in Brazil, most notably *An. darlingi*, *An. albitarsis*, *An. nuneztovari*, *An. aquasalis*, and *An. braziliensis* [33-36]. *An. darlingi* is the most widespread vector and makes the largest contributes to transmission. The mosquito component of the model is calibrated using data from *An. darlingi* in Brazil on mosquito life expectancy, duration of gonotrophic cycle, and the duration of the sporogonic cycle [37]. The assumed parameter values are presented in Table C. Note that since we are not analysing the impact of vector control interventions, model simulations will not be sensitive to the exact choice of entomological parameters.

Table C: Entomological parameters. Parameters are based on *An. darlingi*.

parameter	value
mosquito death rate / life expectancy	1 / 6 day^{-1}
duration of sporogony	8.0 days
dry season proportion	0.2
seasonality shape parameter	2
seasonal peak time	0
human blood index	0.5
endophily (indoor biting)	0.9
proportion of indoor bites	0.2
foraging time	0.68 days
gonotrophic cycle	3 days

3.4 Primaquine adherence

We conducted a literature review for publications measuring adherence to 7 days of prescribed low dose primaquine (0.5 mg/kg/day) among *P. vivax* cases in South American settings. We identified three studies in Brazil [19, 20, 38] one study in Peru [21] and one study in Ecuador [39] published between 2000 and 2015. Two studies including estimates of self-reported adherence to treatment for both *P. falciparum* and *P. vivax* infections in Mato Grosso, Brazil of 83.8% and 77.8% were excluded [40, 41]. The findings of the included studies are summarized in Table M.

Three studies assessed both pill-count and self-reported questionnaires and estimated adherence to primaquine at day 7 at 62.2%, 86.4%, and 66.7%. Only the last and most recent study by Almeida *et al.* assessed the sensitivity and specificity of their questionnaire and was able to estimate an accuracy of 97.1% on the Likert scale questionnaire based on responses to pill-count. Therefore for our model parameters, we assume a 66.7% adherence to 7-day primaquine in Brazil based on this study [19].

3.5 Low CYP2D6 metaboliser prevalence

In 2013, Bennett *et al*. showed a significant association between low CYP2D6 metaboliser activity and primaquine treatment failure [23]. Therefore it is important to evaluate the prevalence of CYP2D6 metaboliser activity, in addition to adherence, when assessing primaquine efficacy. We have compiled relevant studies estimating low CYP2D6 prevalence in Table N.

One issue that arises when estimating low CYP2D6 metaboliser prevalence is the cut-off value for what is considered an intermediate metaboliser. Baird *et al*. have summarized the possible phenotypes [42]. Generally, since the genotype for low CYP2D6 metabolisation lies on two alleles, if both alleles code for a non-functional genotype, the activity score (AS) would correspond to 0.

If both alleles code for an intermediate genotype, the AS would be equal to 1. Some studies categorize low CYP2D6 metaboliser phenotype by including those AS = 1 [24, 25, 27, 43, 44] based on Gaedigk *et al.*'s work [45] while others have excluded them in their reported estimates [23, 46].

In 2017, Gaedigk *et al.* published global estimates of CYP2D6 genotype and phenotype prevalence [26]. When considering complete datasets with all possible allele combinations from individuals in the Americas in this study (Table N), we estimated an average of 28.6% low CYP2D6 prevalence for AS ≤ 1 and 6% considering only AS < 1. Looking at studies estimating CYP2D6 activity among *P. vivax* cases, the prevalence of low CYP2D6 was estimate at 36.4% in AS ≤ 1, which included South East Asian countries that tend to have AS ≤ 1 estimate over 30%.

Among healthy Brazilians, we estimated low CYP2D6 prevalence of AS ≤ 1 at 32.9% and of AS < 1 at 8.1% from Gaedigk *et al.*'s published data provided in the Supplementary Materials. Another Brazilian study also found a similar prevalence of AS < 1 at 9.6% among healthy adults [46]. When considering CYP2D6 prevalence among *P. vivax* cases in the Brazilian population, one estimate found a prevalence 19.5% of AS ≤ 1 [27]. Due to varying levels of uncertainty on the varying definition of low metabolisation, we assumed a low CYP2D6 prevalence of 8.1% for our baseline scenarios and tested the sensitivity of a low (4%) or high (20%) prevalence on results in Section 4.

3.6 Impact of CYP2D6 activity score and *P. vivax* malaria recurrence

When Bennett *et al.* first shed light on the potential impact of CYP2D6 activity scores and *P. vivax* malaria recurrence due to low CYP2D6 metaboliser enzyme for high-dose primaquine (30 mg/day for 14 days) to effectively clear hypnozoites in the liver, their results suggested that for AS < 1 individuals, primaquine was not metabolised effectively [23]. Although a significant association was found, the results have been contested to also include risk among those with an AS = 1. Baird *et al.* were able to show a significant association of relapses in high-dose primaquine (30 mg/day for 14 days) treated *P. vivax* patients with an AS ≤ 1 as compared to those with an AS > 1.5 (OR=9.4, 95% CI 2.1-57.0; $p = 0.001$) [43]. In addition Brasil *et al.* showed that there was a significant increased risk of recurrence in patients with AS ≤ 1 and patients with AS ≥ 1.5 (adjusted RR = 1.89, 95% CI 1.01-3.70; p = 0.049) under low-dose primaquine treatment (15 mg/day for 7 day) [27].

In a large multi-center clinical trial, patients who had poor or intermediate activity (AS ≤ 1) had significantly more recurrences than patients with extensive activity (OR=2.36) under low-dose primaquine (15 mg/day for 14 days) [24]. In addition, St Jean *et al.* were also able to show that intermediate metabolisers (0.5 ≥ AS ≤ 1) had higher odds of relapse than extensive metabolisers (AS ≥ 1.5) with low dose primaquine (15 mg/day for 14 day) (OR=9.18, 95% CI 1-∞) [25].

Impaired CYP2D6 alleles occurred in 95% of therapeutic failures following directly supervised, high-dose primaquine of good quality in subjects followed for a year where reinfection was highly unlikely. Those failures occurred at a rate of 15% among several hundred such treatments [42, 43].

Concerning the impact of CYP2D6 metabolic activity on tafenoquine efficacy, the two previously mentioned randomized multi-center trials did not detect an association with poor or intermediate CYP2D6 metabolisers and clinical relapse for both low-dose tafenoquine (300 mg/day for 1 day) and high-dose tafenoquine (300 mg/day for 2 days) [24, 47]. On the other hand, one study

conducted in mice found that tafenoquine metabolism might not be CYP2D6 dependent [48]. While the surveyed data are certainly consistent with the efficacy of tafenoquine not being affected by low CYP2D6 metabolisation, we do not consider this to be a very high standard of evidence due to the potential for confounding. Therefore, we also implement sensitivity analyses where this assumption is varied.

The association between CYP2D6 activity and the efficacy of 8-aminoquinolines against *P. vivax* hypnozoites is undoubtedly a complex one, with multiple sources of evidence combining to suggest a non-linear dose-response relationship between activity and efficacy. In our main analysis, we make the simplifying assumption that primaquine is not efficacious in individuals with CYP2D6 activity score (AS) < 1, and that primaquine has 71.3% efficacy in individuals with AS ≥ 1. We assume an 8.1% prevalence rate of poor CYP2D6 metabolisers in the Brazilian population. We assume that the efficacy of tafenoquine is not affected by CYP2D6 activity score. Due to the uncertainty of these assumptions, we also consider the impact of varying levels of low CYP2D6 on both primaquine and tafenoquine in extended analyses in Section 4.

3.7 G6PD deficiency prevalence in Brazil

A systematic review reported an average 5.2% G6PD deficiency in Brazil of 9 different states from studies conducted in 2016 and before (Table O) [13]. Co-authors Dr. Wuelton Monteiro, Dr. Marcus Lacerda, and Dr. André Siqueira conducted a prevalence survey of over 14,000 samples in males in 43 municipalities covering six states in the Brazilian Amazon Region in 2018 and estimated an average prevalence of 5.52% (Table D). The municipality-level G6PDd prevalence estimates are shown in Fig F. We calibrated G6PDd prevalence in the model for each municipality for which we have data and assign average state-level prevalence for other municipalities with missing data. Prevalence estimates range from 0% to 10%.

Table D. G6PD deficiency prevalence estimates by state

State	G6PDd prevalence
Overall	5.52%
**Acre**	7.93%
**Amazonas**	3.99%
**Amapá**	5.78%
**Pará**	5.82%
**Rondônia**	5.42%
**Roraima**	3.99%

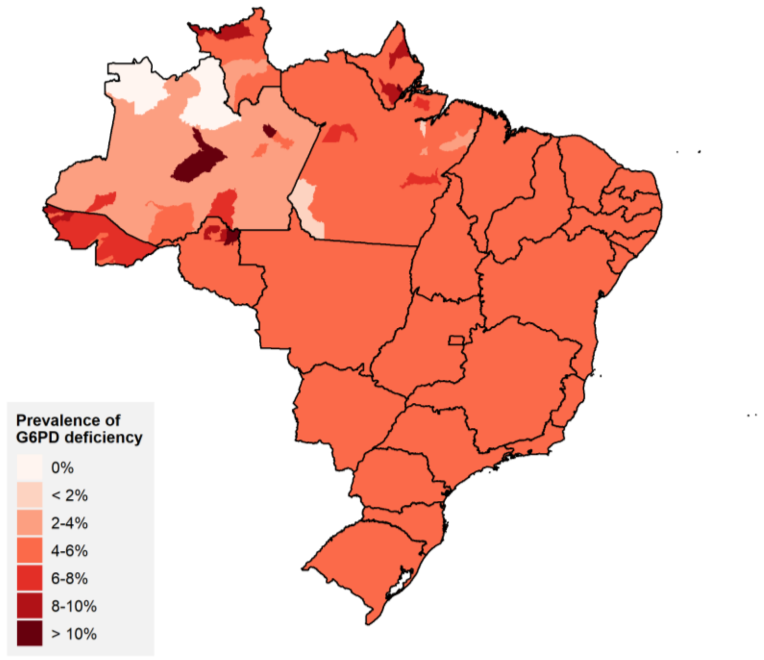

Fig F: Estimated G6PD deficiency prevalence by state or municipality. Estimates are based on the frequency of alleles associated with G6PD deficiency in males. Where the data are sufficient, estimates have been provided on the municipality level. Map generated with shapes files from the R package malariaAtlas.

3.8 Chloroquine resistance and efficacy in clearing blood-stage parasites

Chloroquine resistance has been observed in Brazil. A study assessed resistant parasites to chloroquine mono-therapy in *P. vivax* positive volunteers in Manaus, Brazil, and found that 10.1% of the subjects were confirmed with therapeutic failure at day 28 (*P. vivax* chloroquine resistant) [17]. In another study, the standard treatment regimen of 3-day chloroquine with 7-day primaquine found resistant parasites at day 28 in a total of 5.2% study participants [18]. Due to these differing observations in resistance to chloroquine in Brazil, we will assume 89.9% chloroquine treatment efficacy in clearing blood-stage parasites in patients who are not prescribed primaquine. In patients who are prescribed primaquine, we assume 94.8% efficacy of chloroquine treatment against blood-stage parasites. Note that in patients who are prescribed primaquine but do not adhere to the full 7 day regimen, we also assume 94.8% chloroquine efficacy. In patients that are prescribed both chloroquine and tafenoquine, we assume 100% efficacy against blood-stage parasites due to the very long half-life of tafenoquine.

Extended results assessing a reduction in the prophylactic period of chloroquine are shown in Section 4.10.

3.9 Primaquine efficacy

In order to estimate efficacy of 7-day low dose primaquine treatment in Brazil (3.5 mg/kg total dose), we referred to Brazilian studies of travellers from endemic to non-endemic regions. One study found that 39.6% of treated cases returning from malaria endemic regions relapsed (Pedro 2012) [22]. If we consider only cases who travelled to the Brazilian Amazon Region (n=38), 14 of them relapsed (36.8%). We assume that out of these relapses, 8.1% failed due to due to low

CYP2D6 metabolisers since they were not assessed in the study. Therefore, we assume that low-dose 7-day primaquine efficacy in Brazil is 71.3% (1 - 0.368 + 0.081). This value is also consistent with the proportion of patients free from recurrence seen in clinical trials of 69.6% [24]. In an additional scenario, we assume the potential introduction of a higher efficacy primaquine regimen with 90% efficacy similar to those observed in studies prescribing 5 mg/kg total dose over a course of 14 days [49].

3.10 Tafenoquine efficacy

Phase 3 clinical trials showed comparable efficacy between primaquine and tafenoquine. In patients administered single dose 300 mg tafenoquine, 62.4% (95% CI; 54.9% - 69.0%) remained free from recurrence after 6 months. In patients administered primaquine (15 mg (equivalent to 0.25 mg/kg) daily for 14 days), 69.6% (95% CI; 60.2% - 77.1%) remained free from recurrence after 6 months [24]. This estimate is substantially lower than the DETECTIVE trial of 300mg tafenoquine which reported efficacy against recurrences in Brazil of 89.2% (95% CI; 77% - 95%) [47]. Based on the phase 3 trial data, we assume that primaquine and tafenoquine have equal efficacy. As the efficacy against recurrence at 6 months does not necessarily equal the efficacy against relapses (due to the possibility of reinfection via mosquitoes), we use the value of 71.3% from the Pedro *et al.* study [22]. We also simulated a hypothetical scenario where tafenoquine could be safely given to reach 90% efficacy similar to what was observed in the DETECTIVE trial for a 600 mg dose [50].

3.11 Weekly primaquine

In Brazil, pregnant or lactating women are not prescribed the standard 7-day primaquine regimen. Instead they are prescribed weekly low dose primaquine (0.5 mg/kg) for 8 weeks. Very few studies have assessed the efficacy of this strategy. Only one study found 86.5% efficacy of 0.75 mg/kg primaquine/week for 8 weeks in Pakistan [51]. Furthermore, we were unable to find any data on adherence to this 8-week regimen. We therefore assumed that weekly low dose primaquine for 8 weeks was not efficacious.

4 Extended results

4.1 Radical cure estimates

Based on 2018 case reports, we estimated the number of cases of effective radical cure with the current chloroquine and primaquine treatment per municipality without G6PD testing (Table E, Fig G). Out of a total 217,610 *P. vivax* cases reported in 424 municipalities, 98.2%% were eligible for primaquine. Based on the baseline assumption on adherence and low CYP2D6 metaboliser prevalence, we estimated a municipality-level median of 43.7% (range [19.4%, 43.7%]) effective radical cure equivalent to 92,150 cases. If G6PDd individuals did not fully adhere to the primaquine regimen, we estimated a municipality-level median of 41.2% (range [18.3%, 42.2%]) effective radical cure equivalent to 88,177 cases.

Table E. Estimating radical cure

	Baseline	Considering G6PDd lack of adherence at baseline	Tafenoquine introduction with G6PD testing (S1)
***P. vivax* cases**	217 610 (100%)	217 610 (100%)	217 610 (100%)
**Non-eligible**	4 000 (1.8%)	4 000 (1.8%)	22 883 (10.5%)
**Non-adherence**	71 132 (32.7%)	79 007 (36.4%)	4 942 (2.3%)
**Non-efficacious**	49 143 (22.6%)	46 426 (21.3%)	54 913 (25.2%)
**Effective radical cure**	93 335 (42.9%)	88 177 (40.5%)	134 800 (62.0%)

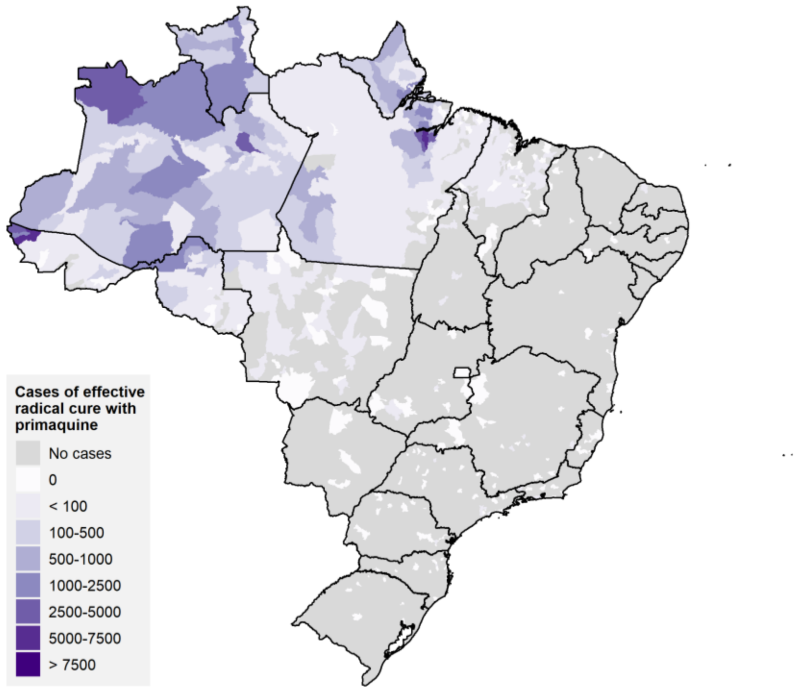

Fig G. Estimated effective radical cure in Brazil in 2018 with primaquine (baseline). Map generated with shapes files from the R package malariaAtlas.

If cases in 2018 were treated with tafenoquine and G6PD testing was introduced (S1), we would expect a 59.0% effective radical cure (range [26.6%, 61.9%]) with an estimated 127,100 treated cases (Table E, Fig H). An additional 35,000 cases could be treated (Fig I).

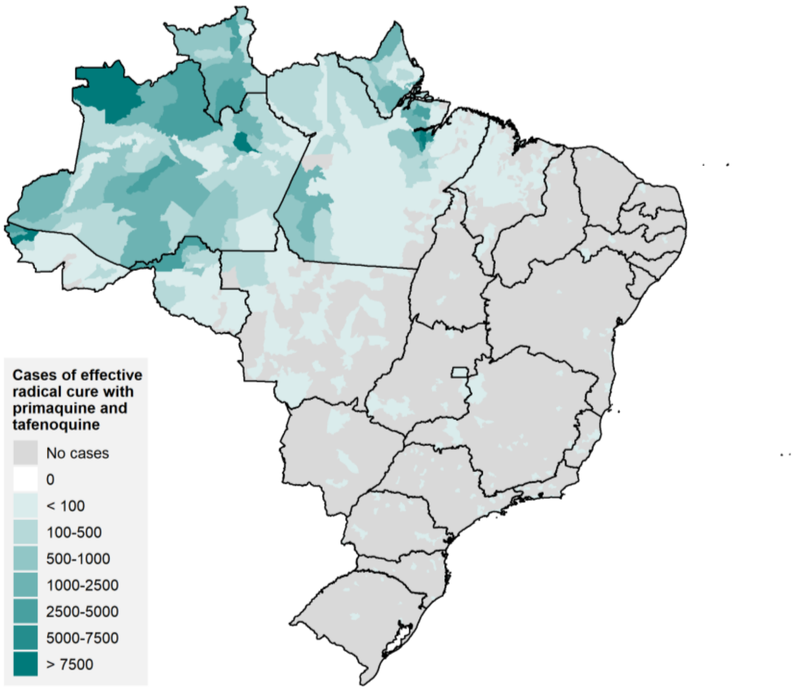

Fig H. Estimated effective radical cure in Brazil in 2018 with primaquine and tafenoquine (S1). Map generated with shapes files from the R package malariaAtlas.

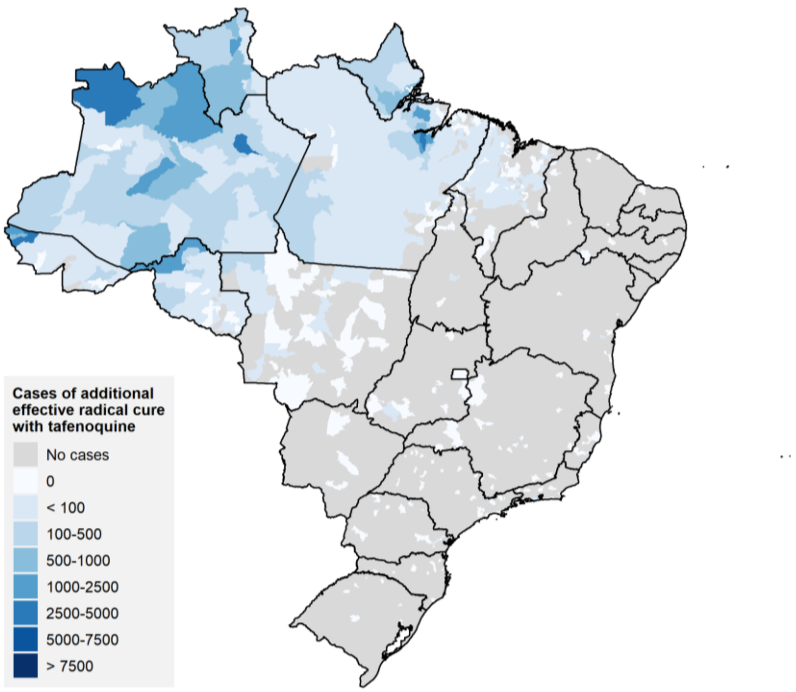

Fig I. Estimated additional cases of effective radical cure in Brazil in 2018 with tafenoquine introduction (S1). Map generated with shapes files from the R package malariaAtlas.

4.2 Asymptomatic infections

The proportion of asymptomatic infections increases with higher transmission intensity. As transmission intensity increases, a higher proportion of sub-patent cases are detected by PCR. These lower levels of parasitemia can go undetected when light microscopy (LM) is the main tool used to detect cases for treatment. As higher intensity of *P. vivax* exposure occurs over time, the population develops levels of immunity that can suppress higher parasitemia all while transmission is still ongoing. These trends explain the differences in the simulation results across different settings such as Itaituba, Pará with an annual parasite incidence (API) or 23 cases per 100 and São Gabriel da Cachoeira, Amazonas, with an API of 267 (Fig J). In lower transmission settings, a higher proportion of cases have clinical disease who are more likely to be detected and treated by a high coverage health care system like that of Brazil (e.g. 18%, Fig J A). As more cases enter the treatment pathway, the effect of radical cure on onwards transmission will be more apparent. As a result, in moderate and high transmission settings, a smaller proportion of cases will be treated out of those who are transmitting *P. vivax* (Fig J B-C). Even if tafenoquine introduction into case management greatly improves the rate of effectively treated cases, the effect size is smaller because only a small percent of cases (i.e. < 15%) are being treated.

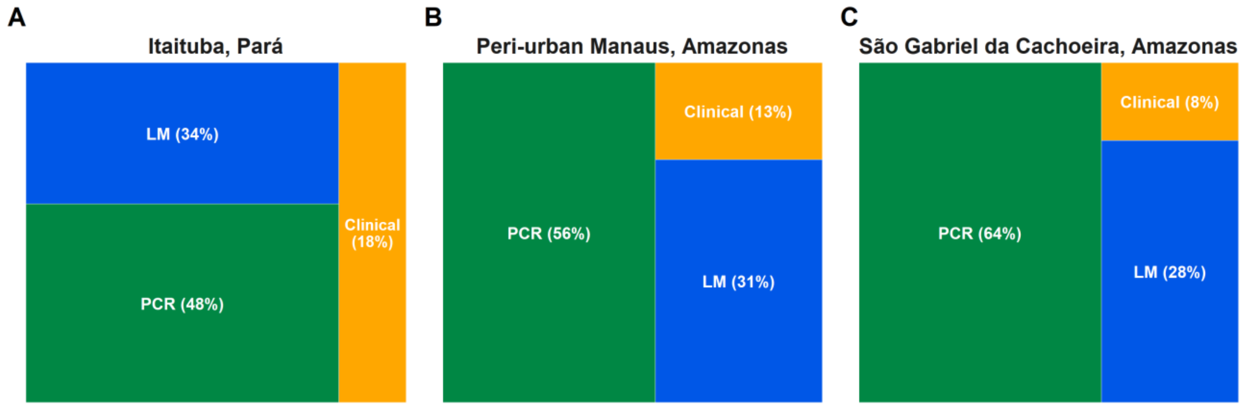

Fig J. Proportion of infections detectable by PCR, light microscopy (LM) and clinical disease. In a representative **(A)** low, **(B)** moderate, and **(C)** high transmission setting.

We observed that in a low transmission setting like Itaituba, Pará, 18% of cases have clinical disease and tafenoquine introduction (S1) would have a more significant impact on transmission reducing incidence from 23 cases to 8.2 cases per 1000 (65% reduction). However, as transmission intensity increases in settings like peri-urban Manaus, Amazonas less than 15% of cases have clinical symptoms and more than 50% have very low parasitemia. In São Gabriel da Cachoeira, where very few cases may be captured by the healthcare system, tafenoquine introduction would only impact transmission in less than 10% of the population and even less because the majority of cases are in children. However, it is important to note that even such small reductions in transmission would result in an important reduction of clinical cases in high transmission settings there is a lower effective size.

4.3 Cases averted, treatment courses and G6PD tests in archetype settings

Table F: *P. vivax* clinical cases averted 5-years post-tafenoquine introduction in low, moderate, and high transmission settings. Assuming incidence and population size calibrated to 2018 data, the total annual clinical cases for each scenario were compared to a baseline scenario with no tafenoquine introduction. We reported the cases averted as the difference in the total cumulative annual cases from 2021 to 2025 in the baseline scenario compared to the tafenoquine introduction scenarios 1-6 over this period. We rounded these values to the nearest 100.

**municipality**	**Itaituba, Pará**	**Manaus, Amazonas**	**São Gabriel da Cachoeira, Amazonas**
exposure setting	occupational	moderate occupational in peri-urban area	peri-domestic
population	101097	100000	44816
cases per 1000 population	23	114	267
annual cases	2360	11400	12030
**modelled scenario**	**Cases averted over 5 years**	**Cases averted over 5 years**	**Cases averted over 5 years**
Scenario 1: standard tafenoquine intervention	3200	8400	4400
Scenario 2: high efficacy tafenoquine intervention	3600	11900	6300
Scenario 3: tafenoquine intervention in young children	2900	13900	11600
Scenario 4: high efficacy tafenoquine intervention in children	4300	18200	15900
Scenario 5: tafenoquine intervention with high pre-existing primaquine adherence	800	2200	2300
Scenario 6: tafenoquine intervention with low pre-existing primaquine adherence	5500	16900	10100

In the intervention strategies considered, the number of courses prescribed is impacted by both transmission intensity, case eligibility, and the impact of different intervention strategies on transmission. Notably, when we compare S1 and S3, a higher efficacy tafenoquine scenario would lead to fewer G6PD tests and fewer courses of tafenoquine being prescribed. This is because the higher efficacy regimen is predicted to lower *P. vivax* transmission more in the community. In some cases, this phenomenon also extends to the situation where children are prescribed tafenoquine (S2). For example, if tafenoquine can be safely prescribed to children >2 years of age, initially more G6PD tests and more courses will be required. However, if expanded case management leads to a reduction in population-level *P. vivax* transmission over the long term, then fewer courses will need to be prescribed. This is also the case in settings with low pre-existing primaquine adherence that would see the most significant impact with tafenoquine introduction and prescribe the least amount of 8-aminoquinolines overall (S6).

Treatment courses and G6PD tests for archetype settings are provided in Table G.

Table G: Treatment courses and G6PD tests summary of model simulations in representative municipalities. Values are cumulative sums during the first 5 years of intervention (from 2021 to 2025).

municipality	Itaituba, Pará				Manaus, Amazonas				São Gabriel da Cachoeira, Amazonas			
exposure setting	occupational				moderate occupational in peri-urban area				peri-domestic			
population	101097				100000				44816			
cases per 1000 population	23				114				267			
**modelled scenario**	**Total courses after 5 yrs**				**Total courses after 5 yrs**				**Total courses after 5 yrs**			
	**CQ**	**PQ**	**TQ**	**G6PD tests**	**CQ**	**PQ**	**TQ**	**G6PD tests**	**CQ**	**PQ**	**TQ**	**G6PD tests**
Baseline	11800	11600	0	0	55100	53100	0	0	57900	55900	0	0
Scenario 1: standard tafenoquine intervention	8400	1900	5200	8200	46400	19000	21600	44600	53100	34500	14400	51000
Scenario 2: 90% efficacy tafenoquine	8000	1900	4700	7700	42900	18400	19000	41200	51300	34000	13200	49300
Scenario 3: tafenoquine for children	8700	700	6500	8400	40800	4100	31400	39100	46000	6100	36000	44000
Scenario 4: 90% efficacy tafenoquine for children	7300	700	5400	7100	36500	3800	27500	34900	41700	5800	32200	39800
Scenario 5: tafenoquine intervention with 90% pre-existing primaquine adherence	10700	2200	6800	10400	52600	20800	25100	50400	55300	34900	15900	53000
Scenario 6: tafenoquine intervention with 30% pre-existing primaquine adherence	6000	1500	3600	5800	37900	17000	16400	36500	47400	32100	11900	45800

4.4 Cases averted at the national level

Table H: *P. vivax* clinical cases averted 5-years post-tafenoquine introduction in 126 simulated settings (excluding Manaus, including per-urban Manaus) and 298 non-simulated settings with 1 to 99 annual cases reported in 2018. For simulated setting: assuming incidence and population size calibrated to 2018 data, the total annual clinical cases for each scenario were compared to a baseline scenario with no tafenoquine introduction. We reported the cases averted as the difference in the total cumulative annual cases from 2021 to 2025 in the baseline scenario compared to the tafenoquine introduction scenarios 1-6 over this period. For non-simulated setting: assuming no change in annual reported *P. vivax* cases across all years and the impact as the median estimated effect size in 2025 per scenario. All values are rounded to the nearest 100.

scenario	Cases averted among modelled scenarios (n = 126)	Non-simulated settings (n = 298)			Total
		Cumulative cases	Median effect size	Estimated averted cases	
Scenario 1: standard tafenoquine intervention	177600	98000	37.5%	36800	214400
Scenario 2: high efficacy tafenoquine intervention	225300	98000	48.1%	47200	272500
Scenario 3: tafenoquine intervention in young children	254100	98000	51.5%	50500	304600
Scenario 4: high efficacy tafenoquine intervention in children	276700	98000	45.0%	44100	320800
Scenario 5: tafenoquine intervention with high pre-existing primaquine adherence	383600	98000	59.6%	58400	442000
Scenario 6: tafenoquine intervention with low pre-existing primaquine adherence	105100	98000	17.7%	17400	122500

4.5 Primaquine adherence

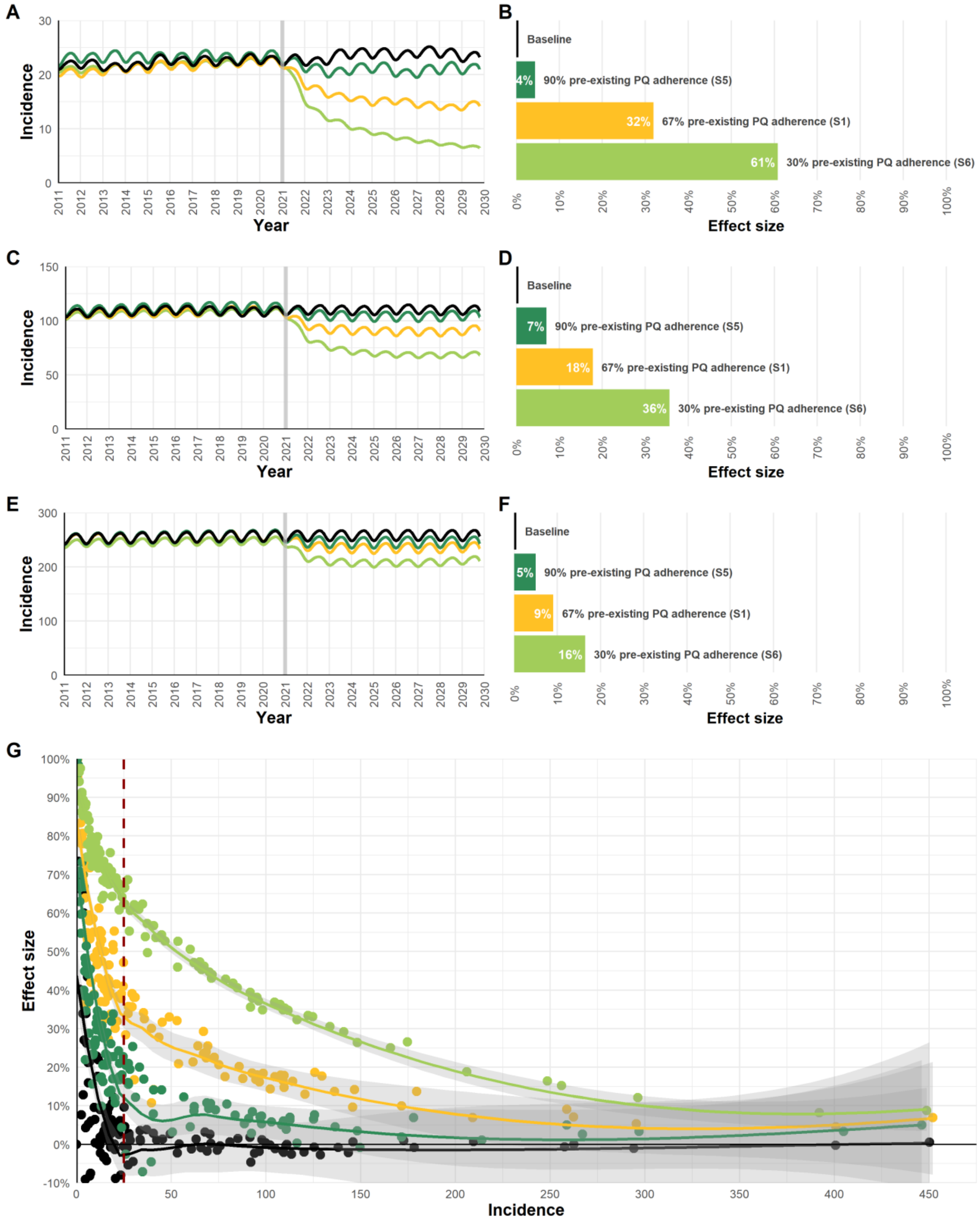

Fig K. Effect of introducing tafenoquine on *P. vivax* incidence in low, moderate, and high transmission settings with varying primaquine adherence. (A) (C) (E) Model simulated *P. vivax* incidence under no intervention and under scenarios 1, 5, and 6 introducing tafenoquine with testing for

G6PDd in 2021 in Itaituba, peri-urban Manaus, and São Gabriel da Cachoeira respectively. Incidence represents the average of 100 independent simulations per scenario with a moving-average smoothing of the data. Incidence was calibrated to clinical cases reported in 2018 per 1000 population. **(B) (D) (F)** The effect size (in %) is reported as the percentage reduction in incidence after 5 years (incidence in 2020 compared to 2025) for S1-4 in Itaituba, peri-urban Manaus, and São Gabriel da Cachoeira respectively. (G) The 5-year post-intervention effect size in all simulate settings. A Loess fitted line and 95% confidence interval bands are shown. For municipalities with an incidence below 25 cases per 1000 population, stochastic fade out in the absence of importation prevents accurate assessment of impact.

4.6 Effect size over time for S1

For our main results, we chose to report the effect size of tafenoquine introduction into case management practices 5-years post-introduction. However, the effect size can be assessed at any point. We show how the percent change intromission across the simulated municipalities under Scenario 1 varies over time (Fig L). At the end of the first year of introduction, tafenoquine is expected to have a median impact of 5.1% (95% UI [-1.8%- 37.1%]) on transmission across all settings and 4.0% (95% UI [2.3%-7.4%] in settings with moderate and high transmission (incidence > 25 cases per 1000) (Fig L A, Table I). An additional median 19.9% and 13.6% gain in impact of transmission is expected in the second year respectively. Impact is not expected to change in settings with very high incidence over time (> 200 cases per 1000 population). Over time, settings will reach a new equilibrium 10-years post-introduction with only small gain in the last 5 years.

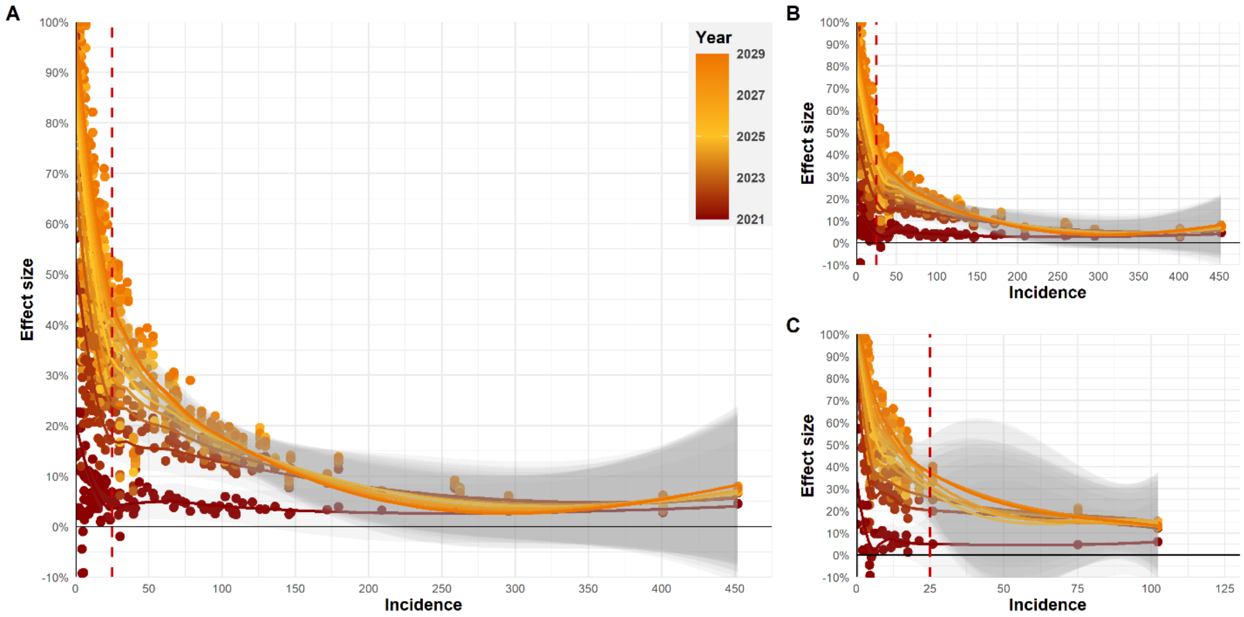

Fig L. The effect size or percent change in transmission post-introduction varies over time. Tafenoquine introduction scenarios were introduced in 2021 and simulated to the end of 2029. Here we show results for S1 for all setting (A), peri-domestic transmission settings (B), and occupational transmission settings (C).

Table I: Median effect size of scenario 1 over time.

Year	Median effect size at year 5		Additional impact over time	
	All settings	incidence > 25 cases per 1000	All settings	incidence > 25 cases per 1000
2021	5.1% [-1.8%-37.1%]	4.0% [2.3%-7.4%]		
2022	19.9% [6.4%-80.5%]	13.6% [5.6%-21.7%]	14.8%	9.6%
2023	27.0% [6.9%-93.6%]	15.5% [5.7%-30.7%]	7.1%	2.0%
2024	32.2% [7.3%-98.6%]	17.2% [5.8%-35.5%]	5.2%	1.7%
2025	37.5% [7%-99.4%]	18.2% [6%-38.7%]	5.3%	1.0%
2026	40.4% [6.9%-100%]	19.4% [5.8%-42.1%]	2.9%	1.2%
2027	44.6% [7%-100%]	19.4% [6.2%-45.4%]	4.2%	0.0%
2028	45.6% [7.1%-100%]	20.1% [6.1%-50.2%]	1.1%	0.7%
2029	47.8% [7.1%-100%]	20.0% [6.4%-51.7%]	2.1%	0.0%

4.7 Effect size summary table for all scenarios 1-10

Refer to section 4.8 and 4.9 for a description of scenarios 7-10.

In S1 we would expect a median 37.5% impact on transmission in all simulated municipalities after 5 years as compared to 18.2% in settings with an incidence > 25 where we have more certainty (Table J). Additional impact can be achieved with the introduction of a higher efficacy dose of tafenoquine safely in adults (S2), in children (S3, S4), and improving primaquine efficacy (S7). Several factors may also give tafenoquine an additional advantage such as low pre-existing primaquine adherence (S6) and significant low CYP2D5 metabolisation in only primaquine (S10).

Table J: Comparing median effect size of scenario 1 and all scenarios across all settings and settings with moderate and high incidence.

modelled scenario	Median effect size at year 5		Compared to S1	
	All settings	incidence per 1000 > 25	All settings	incidence per 1000 > 25
Scenario 1: standard tafenoquine intervention	37.5% [7%-99.4%]	18.2% [6%-38.7%]		
Scenario 2: 90% efficacy tafenoquine	51.5% [9.6%-98.3%]	29.5% [7.5%-52.1%]	14.0%	11.3%
Scenario 3: tafenoquine for children	45.0% [19.2%-89.8%]	32% [13.8%-52.6%]	7.5%	13.8%
Scenario 4: 90% efficacy tafenoquine for children	59.6% [26.6%-99.7%]	43.3% [18.5%-65.9%]	22.1%	25.1%
Scenario 5: tafenoquine intervention with 90% pre-existing primaquine adherence	17.7% [-0.7%-98.1%]	7.1% [-3.8%-22.1%]	-19.8%	-11.1%
Scenario 6: tafenoquine intervention with 30% pre-existing primaquine adherence	66.8% [15.4%-97.1%]	42.3% [10%-62.3%]	29.3%	24.1%
Scenario 7: 90% efficacy tafenoquine and primaquine	55.5% [14.6%-99.6%]	33.6% [10.7%-58%]	18.0%	15.4%
Scenario 8: 8.1% low CYP2D6 prevalence with effect in primaquine and tafenoquine	28.6% [5.7%-89.2%]	15% [4.9%-30.2%]	-8.9%	-3.2%
Scenario 9: 4% low CYP2D6 prevalence with effect in primaquine only	35.3% [6.3%-99.4%]	17.3% [5.4%-35.7%]	-2.2%	-0.9%
Scenario 10: 20% low CYP2D6 prevalence with effect in primaquine only	48.1% [9.1%-96.5%]	25.8% [7.4%-53.4%]	10.6%	7.6%

4.8 Varying assumption of 8-aminoquinoline efficacy

The efficacy of 8-aminoquinolines varies across populations and regimens. In Brazil, we estimated the efficacy of 7-day 3.5mg/kg total dose of primaquine at 71.3% (Section 3.9). Since the regimen for tafenoquine to be introduced in Brazil is a single 300mg dose that administered in clinical trials and shown to have similar efficacy, we assumed that for our standard tafenoquine scenario (S1), tafenoquine would have equivalent efficacy at 71.3% (Section 3.10).

Based on such data, the main advantage of tafenoquine as compared to primaquine is reducing low adherence. In a hypothetical scenario where a high efficacy tafenoquine dose can be safely administered (S2), we would expect an additional impact on transmission of 14.0% as compared to S1 (Fig M). In addition, in South East Asian settings, a 14-day 5 mg/kg primaquine dosing regimen has been shown to have close to 90% efficacy (Section 3.9). In a hypothetical scenario where a similar primaquine regimen is introduced in Brazil along with a higher tafenoquine regimen (S7), we would expect some additional impact. Scenario 7 could lead to a median impact of 55.5% as compared to S2 of 51.5%. In particular, such a regimen would be beneficial to higher transmission settings that have more cases in children and as a result, a higher proportion of

cases prescribed primaquine (Fig M C-F). In lower transmission settings or occupational settings, most cases would already be eligible for tafenoquine and very few cases would be impact by an increase in primaquine efficacy (Fig M A-B).

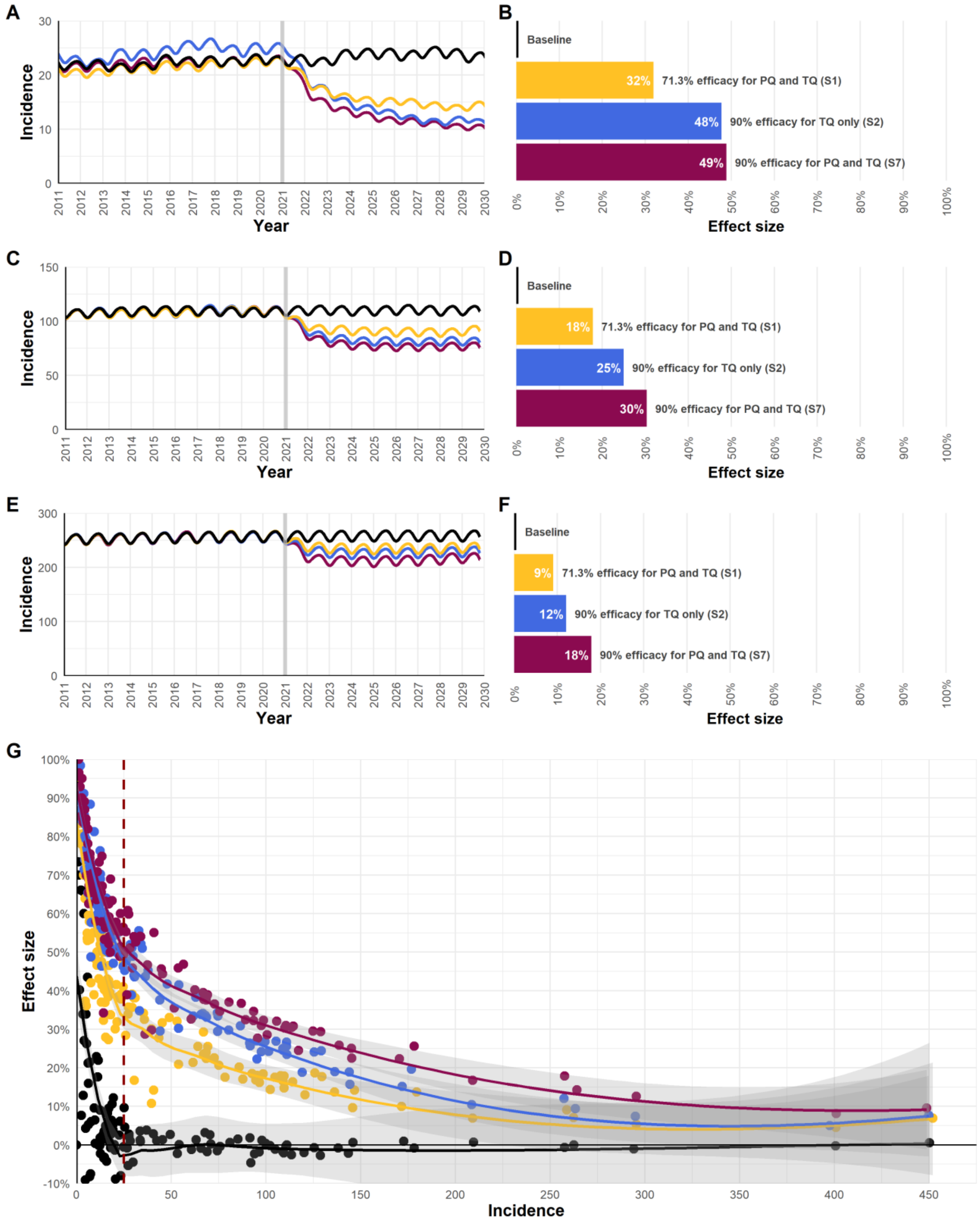

Fig M. Effect of introducing tafenoquine on *P. vivax* incidence in low, moderate, and high transmission settings with varying 8-aminoquinoline efficacy. (A) (C) (E) Model simulated *P. vivax* incidence under no intervention and under scenarios 1, 2, and 7 introducing tafenoquine with testing for G6PDd in 2021 in Itaituba, peri-urban Manaus, and São Gabriel da Cachoeira respectively. Incidence

represents the average of 100 independent simulations per scenario with a moving-average smoothing of the data. Incidence was calibrated to clinical cases reported in 2018 per 1000 population. **(B) (D) (F)** The effect size (in %) is reported as the percentage reduction in incidence after 5 years (incidence in 2020 compared to 2025) for S1-4 in Itaituba, peri-urban Manaus, and São Gabriel da Cachoeira respectively. (G) The 5-year post-intervention effect size in all simulate settings. A Loess fitted line and 95% confidence interval bands are shown. For municipalities with an incidence below 25 cases per 1000 population, stochastic fade out in the absence of importation prevents accurate assessment of impact.

4.9 Varying assumption on low CYP2D6 metabolisation

As previously described in Sections 3.5 and 3.6, there is a high level of uncertainty on the impact of low CYP2D6 metabolisation on 8-aminoquinoline activity and efficacy. The first uncertainty due to lack of sufficient data, is the impact of low CYP2D6 metabolisation of tafenoquine. In S1, we assumed an impact only on primaquine; however, in another scenario (S8), assuming that similar mechanisms of primaquine activation also apply to the drug family of 8-aminoquinolines including tafenoquine. We tested the impact of S8 with an assumed 8.1% low CYP2D6 prevalence on transmission across various settings in Brazil (Fig N). In such a scenario, we would expect the impact of tafenoquine on transmission to reduce by 6.9% (from a median of 37.5% to 28.6%) across all municipalities as compared to S1. In settings with a high burden of disease, this could have significant impact. However, in higher transmission settings where a lower proportion of cases receive tafenoquine (i.e. most cases in children), we would not expect a significant difference (Figs N E-F).

A second level of uncertainty also arises in the classification of low CYP2D6 metabolisation and as a result the prevalence estimate of low metabolisers in Brazil. We assumed a prevalence of 8.1% in S1 and tested the potential parameter uncertainty by simulating scenarios of low CYP2D6 prevalence at 4% (S9) and at 20% (S10). We estimated that a prevalence of 4% with effect only in primaquine would reduce the impact of tafenoquine on transmission 2.2% but generally it would have very little impact on incidence as compared to S1. As the prevalence of low CYP2D6 increases to 20% as shown in some studies in Brazil (Section 3.5, 3.6), we would expect a slightly higher impact for tafenoquine introduction (potential additional increase in impact of 10.6%). If low CYP2D6 does not have an impact of tafenoquine, tafenoquine would be more advantageous in S10 than S1 as compared to baseline; however, this relies on a major assumption that CYP2D6 metabolisation is not an important factor for tafenoquine efficacy, which would overall reduce the impact of any tafenoquine rollout scenario (S8).

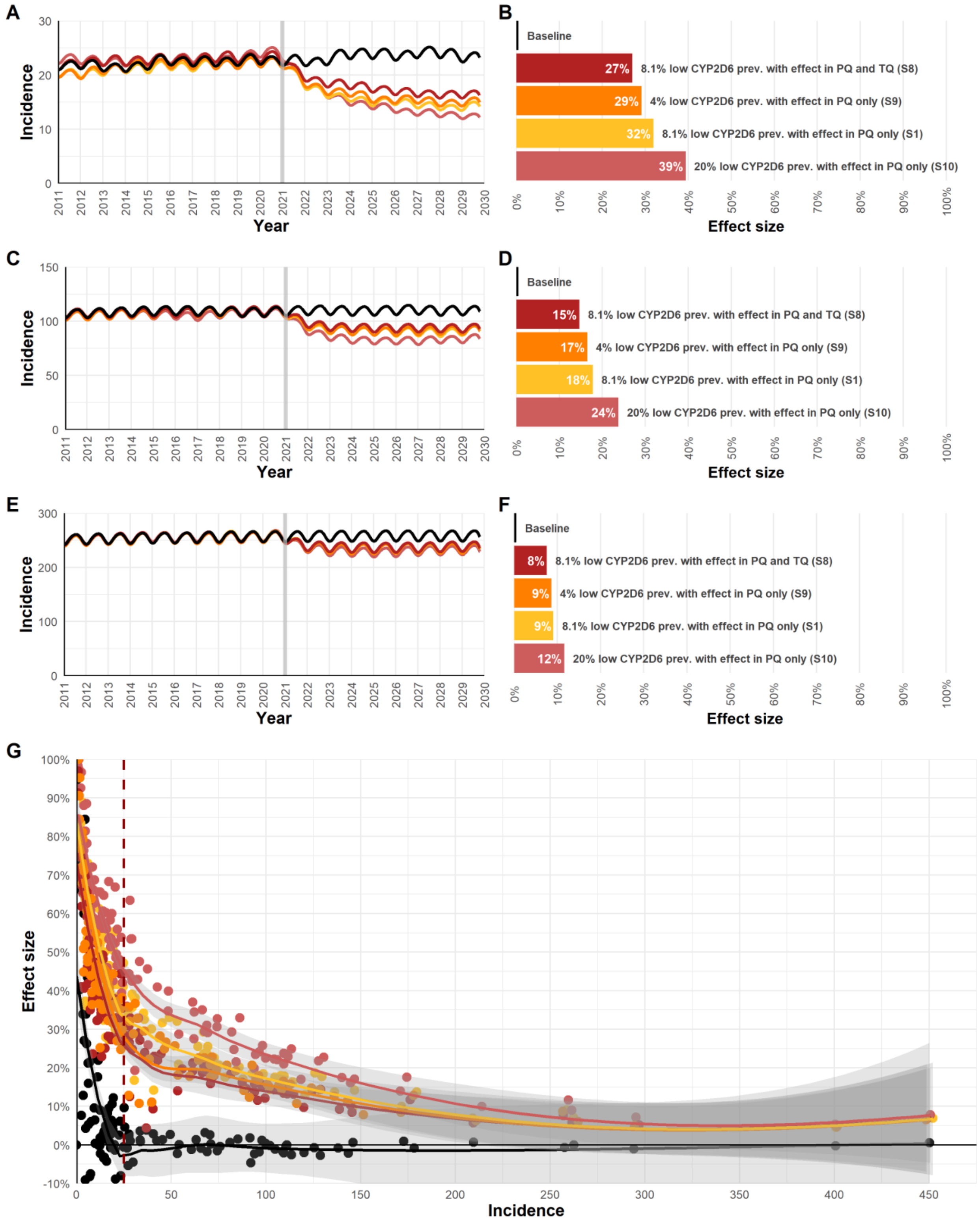

Fig N. Effect of introducing tafenoquine on *P. vivax* incidence in low, moderate, and high transmission settings with low CYP2D6 prevalence and impact on 8-aminoquinolines. (A) (C) (E) Model simulated *P. vivax* incidence under no intervention and under scenarios 1, and 8-10 introducing tafenoquine with testing for G6PDd in 2021 in Itaituba, peri-urban Manaus, and São Gabriel da Cachoeira

respectively. Incidence represents the average of 100 independent simulations per scenario with a moving-average smoothing of the data. Incidence was calibrated to clinical cases reported in 2018 per 1000 population. **(B) (D) (F)** The effect size (in %) is reported as the percentage reduction in incidence after 5 years (incidence in 2020 compared to 2025) for S1-4 in Itaituba, peri-urban Manaus, and São Gabriel da Cachoeira respectively. (G) The 5-year post-intervention effect size in all simulate settings. A Loess fitted line and 95% confidence interval bands are shown. For municipalities with an incidence below 25 cases per 1000 population, stochastic fade out in the absence of importation prevents accurate assessment of impact.

4.10 Variation in blood-stage prophylactic duration

In our model, we assumed that the duration of prophylaxis protecting from new infectious bites in the Brazilian population was 28 days with the 3-day regimen of chloroquine. We assumed little to no chloroquine resistance across all modelled settings. We assessed the impact of chloroquine failure before 28 days set to 14 days of post-treatment prophylaxis with chloroquine across all scenarios (Table K). On average, due to the reduction in the post-blood-stage treatment prophylactic period in these scenarios, we observe a 4-5% increase in the impact of tafenoquine on transmission.

Table K: Comparing the median effect size of all scenarios with CQ post-treatment prophylactic duration of 14 days.

modelled scenario	Median effect size at year 5			
	28-day CQ post-treatment prophylaxis		14-day CQ post-treatment prophylaxis	
	All settings	incidence per 1000 > 25	All settings	incidence per 1000 > 25
Scenario 1: standard tafenoquine intervention	37.5% [7%-99.4%]	18.2% [6%-38.7%]	41.5% [12%-99.7%]	23.8% [10.7%-42.1%]
Scenario 2: 90% efficacy tafenoquine	51.5% [9.6%-98.3%]	29.5% [7.5%-52.1%]	56.8% [13.8%-100%]	33.6% [12.5%-57.2%]
Scenario 3: tafenoquine for children	45.0% [19.2%-89.8%]	32% [13.8%-52.6%]	50.3% [22.8%-94.7%]	35.1% [19%-52.2%]
Scenario 4: 90% efficacy tafenoquine for children	59.6% [26.6%-99.7%]	43.3% [18.5%-65.9%]	62.6% [30%-97.1%]	47.7% [23.2%-64.9%]
Scenario 5: tafenoquine intervention with 90% pre-existing primaquine adherence	17.7% [-0.7%-98.1%]	7.1% [-3.8%-22.1%]	22.3% [4.4%-98%]	11.1% [1.3%-23.9%]
Scenario 6: tafenoquine intervention with 30% pre-existing primaquine adherence	66.8% [15.4%-97.1%]	42.3% [10%-62.3%]	69.4% [21%-97.2%]	47.6% [16.9%-68.6%]
Scenario 7: 90% efficacy tafenoquine and primaquine	55.5% [14.6%-99.6%]	33.6% [10.7%-58%]	57.9% [18.3%-96.9%]	37.9% [15.8%-58.6%]
Scenario 8: 8.1% low CYP2D6 prevalence with effect in primaquine and tafenoquine	28.6% [5.7%-89.2%]	15% [4.9%-30.2%]	34.6% [10.5%-96.5%]	19.8% [10%-34.8%]
Scenario 9: 4% low CYP2D6 prevalence with effect in primaquine only	35.3% [6.3%-99.4%]	17.3% [5.4%-35.7%]	39.3% [10.4%-99.8%]	20.8% [9.8%-40.1%]
Scenario 10: 20% low CYP2D6 prevalence with effect in primaquine only	48.1% [9.1%-96.5%]	25.8% [7.4%-53.4%]	54.0% [13.6%-93.6%]	32.3% [12.6%-53.6%]

5 References

1. White MT, Walker P, Karl S, Hetzel MW, Freeman T, Waltmann A, et al. Mathematical modelling of the impact of expanding levels of malaria control interventions on Plasmodium vivax. Nat Commun. 2018;9(1):3300. doi: 10.1038/s41467-018-05860-8. PubMed PMID: 30120250; PubMed Central PMCID: PMCPMC6097992.

2. Battle KE, Cameron E, Guerra CA, Golding N, Duda KA, Howes RE, et al. Defining the relationship between Plasmodium vivax parasite rate and clinical disease. Malaria J. 2015;14(1):191. doi: 10.1186/s12936-015-0706-3.

3. Moreira CM, Abo-Shehada M, Price RN, Drakeley CJ. A systematic review of sub-microscopic Plasmodium vivax infection. Malaria J. 2015;14:360-. doi: 10.1186/s12936-015-0884-z. PubMed PMID: 26390924.

4. Monteiro W, Karl S, Kuehn A, Almeida A, White M, Vitor-Silva S, et al. Prevalence and Force of Plasmodium Vivax and Plasmodium Falciparum Blood Stage Infection and Associated Clinical Malaria Burden in the Brazilian Amazon. Malaria J. 2020. doi: 10.21203/rs.3.rs-51282/v1.

5. SIVEP-MALÁRIA - Sistema de Informação de Vigilância Epidemiológica - Notificaçáo de Casos [Internet]. Ministério da Saúde. 2019. Available from: http://portalweb04.saude.gov.br/sivep_malaria/.

6. Lacerda MVG, Llanos-Cuentas A, Krudsood S, Lon C, Saunders DL, Mohammed R, et al. Single-Dose Tafenoquine to Prevent Relapse of Plasmodium vivax Malaria. N Engl J Med. 2019;380(3):215-28. doi: 10.1056/NEJMoa1710775. PubMed PMID: 30650322; PubMed Central PMCID: PMCPMC6657226.

7. Watson J, Taylor WRJ, Bancone G, Chu CS, Jittamala P, White NJ. Implications of current therapeutic restrictions for primaquine and tafenoquine in the radical cure of vivax malaria. PLoS Neglected Tropical Diseases. 2018;12(4):e0006440. doi: 10.1371/journal.pntd.0006440.

8. Hounkpatin AB, Kreidenweiss A, Held J. Clinical utility of tafenoquine in the prevention of relapse of Plasmodium vivax malaria: a review on the mode of action and emerging trial data. Infect Drug Resist. 2019;12:553-70. doi: 10.2147/IDR.S151031. PubMed PMID: 30881061.

9. Pal S, Bansil P, Bancone G, Hrutkay S, Kahn M, Gornsawun G, et al. Evaluation of a Novel Quantitative Test for Glucose-6-Phosphate Dehydrogenase Deficiency: Bringing Quantitative Testing for Glucose-6-Phosphate Dehydrogenase Deficiency Closer to the Patient. Am J Trop Med Hyg. 2019;100(1):213-21. doi: 10.4269/ajtmh.18-0612. PubMed PMID: 30350771.

10. Kay A, Kuhl W, Prchal J, Beutler E. The origin of glucose-6-phosphate-dehydrogenase (G6PD) polymorphisms in African-Americans. American Journal of Human Genetics. 1992;50(2):394. PubMed Central PMCID: PMCPMC1682465.

11. Pal S, Bansil P, Bancone G, Hrutkay S, Kahn M, Gornsawun G, et al. Evaluation of a Novel Quantitative Test for Glucose-6-Phosphate Dehydrogenase Deficiency: Bringing

Quantitative Testing for Glucose-6-Phosphate Dehydrogenase Deficiency Closer to the Patient. The American journal of tropical medicine and hygiene. 2019;100(1):213-21. doi: 10.4269/ajtmh.18-0612. PubMed PMID: 30350771.

12. Auton A, Abecasis GR, Altshuler DM, Durbin RM, Abecasis GR, Bentley DR, et al. A global reference for human genetic variation. Nature. 2015;526(7571):68-74. doi: 10.1038/nature15393.

13. Dombrowski JG, Souza RM, Curry J, Hinton L, Silva NRM, Grignard L, et al. G6PD deficiency alleles in a malaria-endemic region in the Western Brazilian Amazon. Malaria J. 2017;16(1):253-. doi: 10.1186/s12936-017-1889-6. PubMed PMID: 28619120.

14. Vizzi E, Bastidas G, Hidalgo M, Colman L, Pérez HA. Prevalence and molecular characterization of G6PD deficiency in two Plasmodium vivax endemic areas in Venezuela: predominance of the African A-(202A/376G) variant. Malaria J. 2016;15:19-. doi: 10.1186/s12936-015-1069-5. PubMed PMID: 26753754.

15. Minucci A, Moradkhani K, Hwang MJ, Zuppi C, Giardina B, Capoluongo E. Glucose-6-phosphate dehydrogenase (G6PD) mutations database: Review of the “old” and update of the new mutations. Blood Cells, Molecules, and Diseases. 2012;48(3):154-65. doi: https://doi.org/10.1016/j.bcmd.2012.01.001.

16. Gómez-Manzo S, Marcial-Quino J, Vanoye-Carlo A, Serrano-Posada H, Ortega-Cuellar D, González-Valdez A, et al. Glucose-6-Phosphate Dehydrogenase: Update and Analysis of New Mutations around the World. Int J Mol Sci. 2016;17(12):2069. doi: 10.3390/ijms17122069. PubMed PMID: 27941691.

17. de Santana Filho FS, Arcanjo ARdL, Chehuan YM, Costa MR, Martinez-Espinosa FE, Vieira JL, et al. Chloroquine-resistant Plasmodium vivax, Brazilian Amazon. Emerg Infect Dis. 2007;13(7):1125-6. doi: 10.3201/eid1307.061386. PubMed PMID: 18214203.

18. Marques MM, Costa MRF, Santana Filho FS, Vieira JLF, Nascimento MTS, Brasil LW, et al. Plasmodium vivax chloroquine resistance and anemia in the western Brazilian Amazon. Antimicrob Agents Chemother. 2014;58(1):342-7. Epub 10/28. doi: 10.1128/AAC.02279-12. PubMed PMID: 24165179.

19. Almeida ED, Rodrigues LCS, Vieira JLF. Estimates of adherence to treatment of vivax malaria. Malaria J. 2014;13:321-. doi: 10.1186/1475-2875-13-321. PubMed PMID: 25127886.

20. Pereira EA, Ishikawa EAY, Fontes CJF. Adherence to Plasmodium vivax malaria treatment in the Brazilian Amazon Region. Malaria J. 2011;10:355-. doi: 10.1186/1475-2875-10-355. PubMed PMID: 22165853.

21. Grietens KP, Soto V, Erhart A, Ribera JM, Toomer E, Tenorio A, et al. Adherence to 7-day primaquine treatment for the radical cure of P. vivax in the Peruvian Amazon. Am J Trop Med Hyg. 2010;82(6):1017-23. doi: 10.4269/ajtmh.2010.09-0521. PubMed PMID: 20519594.

22. Pedro RS, Guaraldo L, Campos DP, Costa AP, Daniel-Ribeiro CT, Brasil P. Plasmodium vivax malaria relapses at a travel medicine centre in Rio de Janeiro, a non-endemic area in Brazil. Malaria J. 2012;11(1):245. doi: 10.1186/1475-2875-11-245.

23. Bennett JW, Pybus BS, Yadava A, Tosh D, Sousa JC, McCarthy WF, et al. Primaquine Failure and Cytochrome P-450 2D6 in Plasmodium vivax Malaria. N Engl J Med. 2013;369(14):1381-2. doi: 10.1056/NEJMc1301936. PubMed PMID: 24088113.

24. Lacerda MVG, Llanos-Cuentas A, Krudsood S, Lon C, Saunders DL, Mohammed R, et al. Single-Dose Tafenoquine to Prevent Relapse of Plasmodium vivax Malaria. N Engl J Med. 2019;380(3):215-28. doi: 10.1056/NEJMoa1710775. PubMed PMID: 30650322; PubMed Central PMCID: PMCPMC6657226.

25. St Jean PL, Xue Z, Carter N, Koh GCKW, Duparc S, Taylor M, et al. Tafenoquine treatment of Plasmodium vivax malaria: suggestive evidence that CYP2D6 reduced metabolism is not associated with relapse in the Phase 2b DETECTIVE trial. Malaria J. 2016;15:97-. doi: 10.1186/s12936-016-1145-5. PubMed PMID: 26888075.

26. Gaedigk A, Sangkuhl K, Whirl-Carrillo M, Klein T, Leeder JS. Prediction of CYP2D6 phenotype from genotype across world populations. Genet Med. 2017;19(1):69-76. Epub 07/07. doi: 10.1038/gim.2016.80. PubMed PMID: 27388693.

27. Brasil LW, Rodrigues-Soares F, Santoro AB, Almeida ACG, Kühn A, Ramasawmy R, et al. CYP2D6 activity and the risk of recurrence of Plasmodium vivax malaria in the Brazilian Amazon: a prospective cohort study. Malaria J. 2018;17(1):57. doi: 10.1186/s12936-017-2139-7.

28. Lover AA, Coker RJ. Quantifying effect of geographic location on epidemiology of Plasmodium vivax malaria. Emerg Infect Dis. 2013;19(7):1058-65. doi: 10.3201/eid1907.121674. PubMed PMID: 23763820.

29. Battle KE, Karhunen MS, Bhatt S, Gething PW, Howes RE, Golding N, et al. Geographical variation in Plasmodium vivax relapse. Malaria J. 2014;13(1):144. doi: 10.1186/1475-2875-13-144.

30. Daher A, Silva JCAL, Stevens A, Marchesini P, Fontes CJ, Ter Kuile FO, et al. Evaluation of Plasmodium vivax malaria recurrence in Brazil. Malaria J. 2019;18(1):18-. doi: 10.1186/s12936-019-2644-y. PubMed PMID: 30670020.

31. White MT, Shirreff G, Karl S, Ghani AC, Mueller I. Variation in relapse frequency and the transmission potential of Plasmodium vivax malaria. Proc Biol Sci. 2016;283(1827):20160048-. doi: 10.1098/rspb.2016.0048. PubMed PMID: 27030414.

32. Simões LR, Alves Jr ER, Ribatski-Silva D, Gomes LT, Nery AF, Fontes CJF. Factors associated with recurrent Plasmodium vivax malaria in Porto Velho, Rondônia State, Brazil, 2009. Cadernos de Saúde Pública. 2014;30:1403-17.

33. Sinka ME, Rubio-Palis Y, Manguin S, Patil AP, Temperley WH, Gething PW, et al. The dominant Anopheles vectors of human malaria in the Americas: occurrence data, distribution maps and bionomic précis. Parasites & Vectors. 2010;3(1):72. doi: 10.1186/1756-3305-3-72.

34. Tadei WP, Dutary Thatcher B. Malaria vectors in the Brazilian Amazon: Anopheles of the subgenus Nyssorhynchus. Revista do Instituto de Medicina Tropical de São Paulo. 2000;42:87-94.

35. Martins-Campos KM, Pinheiro WD, Vítor-Silva S, Siqueira AM, Melo GC, Rodrigues IC, et al. Integrated vector management targeting Anopheles darlingi populations decreases malaria incidence in an unstable transmission area, in the rural Brazilian Amazon. Malaria J. 2012;11:351-. doi: 10.1186/1475-2875-11-351. PubMed PMID: 23088224.

36. Barbosa LMC, Souto RNP, dos Anjos Ferreira RM, Scarpassa VM. Behavioral patterns, parity rate and natural infection analysis in anopheline species involved in the transmission of malaria in the northeastern Brazilian Amazon region. Acta Tropica. 2016;164:216-25. doi: https://doi.org/10.1016/j.actatropica.2016.09.018.

37. de Barros FSM, Honório NA, Arruda ME. Survivorship of Anopheles darlingi (Diptera: Culicidae) in relation with malaria incidence in the Brazilian Amazon. PLoS One. 2011;6(8):e22388-e. Epub 08/08. doi: 10.1371/journal.pone.0022388. PubMed PMID: 21857927.

38. Osorio-de-Castro CGS, Suárez-Mutis MC, Miranda ES, Luz TCB. Dispensing and determinants of non-adherence to treatment for non complicated malaria caused by Plasmodium vivax and Plasmodium falciparum in high-risk municipalities in the Brazilian Amazon. Malaria J. 2015;14:471-. doi: 10.1186/s12936-015-0998-3. PubMed PMID: 26611324.

39. Yépez MC, Zambrano D, Carrasco F, Yépez RF. Factores asociados con el incumplimiento del tratamiento antipalúdico en pacientes ecuatorianos. Revista Cubana de Medicina Tropical. 2000;52(2):81-9.

40. Taisa Guimarães de S, Annelita Almeida Oliveira R, Rosemeiry Capriata de Souza A, Cor Jesus Fernandes F, Rita Graziela F, Priscilla Uiara do C. Malaria knowledge and treatment adherence in a Brazilian Amazon community. The Journal of Infection in Developing Countries. 2016;10(11). doi: 10.3855/jidc.7129.

41. Duarte EC, Gyorkos TW. Self-reported compliance with last malaria treatment and occurrence of malaria during follow-up in a Brazilian Amazon population. Tropical Medicine & International Health. 2003;8(6):518-24. doi: 10.1046/j.1365-3156.2003.01042.x.

42. Baird JK, Battle KE, Howes RE. Primaquine ineligibility in anti-relapse therapy of Plasmodium vivax malaria: the problem of G6PD deficiency and cytochrome P-450 2D6 polymorphisms. Malaria J. 2018;17(1):42-. doi: 10.1186/s12936-018-2190-z. PubMed PMID: 29357870.

43. Baird JK, Louisa M, Noviyanti R, Ekawati L, Elyazar I, Subekti D, et al. Association of Impaired Cytochrome P450 2D6 Activity Genotype and Phenotype With Therapeutic Efficacy of Primaquine Treatment for Latent Plasmodium vivax Malaria. JAMA Network Open. 2018;1(4):e181449-e. doi: 10.1001/jamanetworkopen.2018.1449.

44. Daher A, Aljayyoussi G, Pereira D, Lacerda MVG, Alexandre MAA, Nascimento CT, et al. Pharmacokinetics/pharmacodynamics of chloroquine and artemisinin-based combination therapy with primaquine. Malaria J. 2019;18(1):325. doi: 10.1186/s12936-019-2950-4.

45. Gaedigk A, Simon S, Pearce R, Bradford L, Kennedy M, Leeder J. The CYP2D6 Activity Score: Translating Genotype Information into a Qualitative Measure of Phenotype. Clinical Pharmacology & Therapeutics. 2008;83(2):234-42. doi: 10.1038/sj.clpt.6100406.

46. Friedrich DC, Genro JP, Sortica VA, Suarez-Kurtz G, de Moraes ME, Pena SDJ, et al. Distribution of CYP2D6 alleles and phenotypes in the Brazilian population. PLoS One. 2014;9(10):e110691-e. doi: 10.1371/journal.pone.0110691. PubMed PMID: 25329392.

47. Llanos-Cuentas A, Lacerda MVG, Hien TT, Vélez ID, Namaik-larp C, Chu CS, et al. Tafenoquine versus Primaquine to Prevent Relapse of Plasmodium vivax Malaria. New England Journal of Medicine. 2019;380(3):229-41. doi: 10.1056/NEJMoa1802537. PubMed PMID: 30650326.

48. Marcsisin SR, Sousa JC, Reichard GA, Caridha D, Zeng Q, Roncal N, et al. Tafenoquine and NPC-1161B require CYP 2D metabolism for anti-malarial activity: implications for the 8-aminoquinoline class of anti-malarial compounds. Malaria J. 2014;13:2-. doi: 10.1186/1475-2875-13-2. PubMed PMID: 24386891.

49. Commons RJ, Simpson JA, Thriemer K, Humphreys GS, Abreha T, Alemu SG, et al. The effect of chloroquine dose and primaquine on Plasmodium vivax recurrence: a WorldWide Antimalarial Resistance Network systematic review and individual patient pooled meta-analysis. Lancet Infect Dis. 2018;18(9):1025-34. doi: 10.1016/S1473-3099(18)30348-7.

50. Llanos-Cuentas A, Lacerda MV, Rueangweerayut R, Krudsood S, Gupta SK, Kochar SK, et al. Tafenoquine plus chloroquine for the treatment and relapse prevention of Plasmodium vivax malaria (DETECTIVE): a multicentre, double-blind, randomised, phase 2b dose-selection study. Lancet. 2014;383(9922):1049-58. doi: https://doi.org/10.1016/S0140-6736(13)62568-4.

51. Leslie T, Mayan I, Mohammed N, Erasmus P, Kolaczinski J, Whitty CJM, et al. A randomised trial of an eight-week, once weekly primaquine regimen to prevent relapse of plasmodium vivax in Northwest Frontier Province, Pakistan. PLoS One. 2008;3(8):e2861-e. doi: 10.1371/journal.pone.0002861. PubMed PMID: 18682739.

52. Ley B, Alam MS, O'Donnell JJ, Hossain MS, Kibria MG, Jahan N, et al. A Comparison of Three Quantitative Methods to Estimate G6PD Activity in the Chittagong Hill Tracts, Bangladesh. PLoS One. 2017;12(1):e0169930. doi: 10.1371/journal.pone.0169930.

53. Roca-Feltrer A, Khim N, Kim S, Chy S, Canier L, Kerleguer A, et al. Field trial evaluation of the performances of point-of-care tests for screening G6PD deficiency in Cambodia. PLoS One. 2014;9(12):e116143-e. doi: 10.1371/journal.pone.0116143. PubMed PMID: 25541721.

54. Satyagraha AW, Sadhewa A, Elvira R, Elyazar I, Feriandika D, Antonjaya U, et al. Assessment of Point-of-Care Diagnostics for G6PD Deficiency in Malaria Endemic Rural Eastern Indonesia. PLoS Neglected Tropical Diseases. 2016;10(2):e0004457. doi: 10.1371/journal.pntd.0004457. PubMed PMID: 26894297; PubMed Central PMCID: PMCPMC4760930.

55. Algur N, Avraham I, Hammerman C, Kaplan M. Quantitative Neonatal Glucose-6-Phosphate Dehydrogenase Screening: Distribution, Reference Values, and Classification by Phenotype. The Journal of Pediatrics. 2012;161(2):197-200. doi: https://doi.org/10.1016/j.jpeds.2012.02.045.

6 Appendix

Table L. Studies reporting G6PD activity scores

Study year	Location	Study population	Males + Females	G6PD test	Reported G6PDd prevalence	Model estimate	Ref.
2015-2016	Bangladesh	Healthy adults	398+599	CareStart G6PD Biosensor (G6PD RDT, AccessBio, USA)	8.9%	4.29%	[52]
2013	Cambodia	Healthy adults	451+487	CareStart G6PD (G6PD RDT, AccessBio, USA)	7.9%	12.71%	[53]
2014	Indonesia	Healthy adults	257+328	CareStart G6PD (G6PD RDT, AccessBio, USA)	7.2%	6.65%	[54]
	Israel	Neonates	1502+1298	Quantitative G6PD testing (Sentinal Diagnostics, Milan, Italy)	16.2%	13.28%	[55]

Table M. Studies assessing adherence of P. vivax cases to 7-day primaquine in South America

Study period	Location	Population size	Population characteristics	Malaria type and treatment	Primaquine adherence from questionnaires	Primaquine adherence from pill-count	Primaquine overall adherence	Ref.
	Quinindé, Esmeraldas, Ecuador	126		*P. vivax*; 3-day chloroquine + 7-day primaquine		58.1%		[39]
2005-2007	San Juan, Iquitos, Peru	1072 patients from database of which 185 interviewed	Randomly selected patient from healthcare database with confirmed *P. vivax* in last year	*P. vivax*; 3-day chloroquine + 7-day primaquine	79.8% (of those interviewed)	77.9%	62.2% (0.779 – 0.779*(1-0.798))	[21]
2008-2009	Alenquer, Goianesia do Pará, Itaituba, Maraba, and Santarem, Pará, Brazil	280	> 6 months old patients without mixed or severe malaria or history of allergy to treatment	*P. vivax*; 3-day chloroquine + 7-day primaquine	96.1%	90.4%	86.4% (1 – (1-0.961) – (1-0.904)	[20]
2007	Cruzeiro do Sul, Rodrigues Alves in Acre, Manaus, Presidente Figueiredo in Amazonas, Porto Velho, Ariquemes in Rôndonia, Brazil	134	> 15 year olds with confirmed mild malaria	*P. vivax*; 3-day chloroquine + 7-day primaquine	91.0% (assessed at day 5)	82.1% (assessed at day 5)		[38]
2012	Anajas, Pará, Brazil	135	18-52 years olds without mixed or severe malaria, parasite density over 5%, G6PDd, and prior anti-malarial drugs treatment	*P. vivax*; 3-day chloroquine + 7-day primaquine	72.7% dichotomous; 63.8% Likert; 65.2% Liker dichotomized; 68.9% overall dichotomous	71.1%	66.7% (Likert scale with pill count; 97.1% accuracy)	Table 6 [19]

Table N. Studies assessing CYP2D6 metaboliser prevalence by phenotype. AS refers to the predicted activity score.

Study year	Study population and location	Poor CYP2D6 phenotype AS = 0%	Intermediate CYP2D6 phenotype AS = 0.5% - <1%	Intermediate CYP2D6 phenotype AS = 0.5% - ≤1%	Low CYP2D6 phenotype AS < 1%	Low CYP2D6 phenotype AS ≤1%	Normal metaboliser phenotype AS ≥1.5%	Ref.
2008	1020 healthy adults, Brazil	2.6% (26/1020)	7.1%% (72/1020)		9.6% (98/1020)		87.2% (889/1020) NA excluded, 1% included	Table 6 & Table S1 [46]
2012	25 *P.vivax* patients that relapsed, Thailand	4% (1/25)	12% (3/25)	40% (10/25)	16% (4/25)	44% (11/25)	56% (14/25)<br><br>84% (21/25) including those with 1% AS	Table 1 [23]
2011-2013	199 *P.vivax* patients, Peru, Thailand, India	0.5% (1/199)<br>0.9% (Peru)<br>0% (Thailand)		19.1% (38/199)<br>10.5% (Peru)<br>31.7% (Thailand)		19.6% (39/199)<br>11.4% (Peru)<br>31.7% (Thailand)	80.4% (160/199)<br>88.6% (Peru)<br>68.3% (Thailand)<br>100% (India)	Table 2 [25]
2015	Brazilians with complete data	2.9%	5.3%	30%	8.1%	32.9%	67.1%	Supplementary Materials [26]
	190 *P.vivax* patients, Manaus, Brazil					19.5% (37/190)	80.5% (153/199)	Table 2 [27]
2014	57 *P.vivax* patients, Indonesia	1.8% (1/57)		54.4% (31/57)		56.1% (32/57)	43.9% (25/57)	Table 2 [43]
2014-2016	504 *P.vivax* patients with AS, Ethiopia, Peru, Brazil, Cambodia, Thailand, Philippines	1.6% (8/504)		24.4% (123/504)		26.0% (131/504)	74.0% (373/504)	Table 1 [24]

Table O. Studies estimating G6PD deficiency prevalence in Brazil

Study year	Location	Study population	Prevalence (mean)	Reference
2000 - 2016	Brazil (9 states)		5.20%	Table 3 [13]
2007; 2016	Acre	Children (<10 years of age); adult males	6.68%	Table 3 [13]
2006; 2010	Amazonas	Ismail Aziz Community; adult males	3.75%	Table 3 [13]
2000	Bahia	Neonates	9.95%	Table 3 [13]
2011	Piauí	Adult population	7.40%	Table 3 [13]
2003; 2005; 2005; 2007	Rio Grande do Sul	Newborns; suspected G6PD deficient; patients with jaudice; newborns (50% presenting jaudice)	7.83%	Table 3 [13]
2004	Rondônia	Suspected malaria cases	2.54%	Table 3 [13]
2004	São Paulo	Adult males-blood donors; adult males; male children; adult males-blood donors	2.78%	Table 3 [13]
