# Supplementary material for: Estimated impact of tafenoquine for Plasmodium vivax control and elimination in Brazil: A modelling study
Source: PLoS Med. 2021 Apr 23;18(4):e1003535. doi: 10.1371/journal.pmed.1003535 (PMC8064585; doi:10.1371/journal.pmed.1003535)
Supplement: Attachment — Submitted filename: Response to reviewers.pdf [file pmed.1003535.s002.pdf]

Malaria: Parasites and Hosts
Dept. of Parasites and Insect Vectors
Institut Pasteur
25-28 Rue du Dr Roux
Paris 75015, France

Dr Narimane Nekkab
narimane.nekkab@pasteur.fr

Dr Michael White
michael.white@pasteur.fr

6th November 2020

The Editor
PLOS Medicine

Dear Editor,

We would like to re-submit the enclosed revised manuscript – "Tafenoquine for *Plasmodium vivax* elimination: a modelling case study of Brazil" – for consideration for publication in PLOS Medicine for the call for *P. vivax* malaria research studies. Following your comments and those of the three reviewers, we have carefully considered each point made and, when appropriate, made modifications to the manuscript or supporting information. The manuscript with track changes is enclosed in the resubmission.

During our revision process, we updated one of our model parameters. The duration of chloroquine prophylaxis was parameterized as 14 days, which we updated to 28 days to better reflect the Brazilian situation with low levels of chloroquine resistance. With this update, we re-ran all model simulations (100 iterations for each scenario 0-10 for each 127 settings). While this had a minor impact on our estimates, with the effect size decreasing by approximately 4% across all settings and scenarios, our main conclusions did not change. These additional modifications include changes in values in the text, tables, and figures. The original estimates with the 14 day duration of chloroquine prophylaxis represent scenarios with high levels of chloroquine resistance, and thus these results have been retained in a new Supplementary Information Section 4.10 for comparison.

Enclosed in this letter is our point-by-point responses to each suggested revision. For each point, we respond in grey and show modifications to the text in red. We look forward to your response.

Yours sincerely,

Dr Narimane Nekkab & Dr Michael White, on behalf of the authors

Revisions

Requests from the editors:

*In the last sentence of the Abstract Methods and Findings section, please add a sentence/note about any key limitation(s) of the study's methodology.

We acknowledge that in our initial submission of the manuscript we overlooked the limitations of our work. After reflecting on how certain model assumptions and methodological approaches may not fully reflect real-world dynamics, we have identified several key limitations of our work. We have now addressed these issues in both the abstract (lines 36-38) and the discussion (lines 503-516).

*Please note the author guidelines for the "Author summary" structure - https://journals.plos.org/plosmedicine/s/revising-your-manuscript#loc-author-summary - each subsection should be formatted as 2-3 single sentence bullet points for each of the sections (ie not paragraph text). See here for an example - https://journals.plos.org/plosmedicine/article?id=10.1371/journal.pmed.1003318

We overlooked formatting the author summary appropriately in our initial submission and thank the editor for the note. We have reformatted the summary as 2-3 single sentence bullet points for each section in lines 45-88.

*On page 7, the methods note the SIVEP database for certain parameter estimates (and this is also noted in the supplementary material pdf) - can a citation, web link or reference be added for the database so that readers can ascertain where the underlying data can be found? The same is true for other databases noted as the source for parameters fed into the model.

We were granted access to the SIVEP database by the Brazilian Health Ministry. Data was extracted from an online portal by Brazilian co-authors. We have added reference [27] with the website link to ascertain where the data source.

[27] SIVEP-MALÁRIA - Sistema de Informação de Vigilância Epidemiológica - Notificaçáo de Casos [Internet]. Ministério da Saúde. 2019. Available from: http://portalweb04.saude.gov.br/sivep_malaria/.

Other data sources are now also properly referenced and/or data is now shared in the Supplementary Information. Please see next comment for details.

*On page 8, the paper notes the data source for a point about intra-municipality heterogeneity in transmission with reference to "Monteiro et al., manuscript in preparation". If that paper (the Monteiro one) isn't published by the time that the current PLOS Medicine submission might be ready for acceptance, assuming it fully passes editorial and peer review, then the unpublished paper can't really be cited as the support for a particular claim or assumption - because readers will not be able to go and check it. We realise this is challenging, but the authors will need to cite some published or accepted article or database as the source for the claim. (see below). If this isn't possible then it is possible to convert the intext note to a "personal communication" instead (and as stated in our author guidelines the authors need to be prepared to support, if requested, the pers.comm. with a letter from the relevant individual).

We agree that this is an important point to rectify for readers and as such, we have updated the reference to support our claim. A pre-print version of the article has recently become available. We have added this reference [29] to both the methods section in the revised manuscript (line 178).

Here is the new full citation:
[29] Monteiro W, Karl S, Kuehn A, Almeida A, White M, Vitor-Silva S, et al. Prevalence and Force of Plasmodium Vivax and Plasmodium Falciparum Blood Stage Infection and Associated Clinical Malaria Burden in the Brazilian Amazon. Malar J. 2020. doi: 10.21203/rs.3.rs-51282/v1.

******
https://journals.plos.org/plosmedicine/s/submission-guidelines#loc-reference-style -

Do not cite the following sources in the reference list:

>Unavailable and unpublished work, including manuscripts that have been submitted but not yet accepted (e.g., “unpublished work,” “data not shown”). Instead, include those data as supplementary material or deposit the data in a publicly available database.
>Personal communications (these should be supported by a letter from the relevant authors but not included in the reference list)

All references to data from personal communications have been replaced with references in the main text (see previous comment). All datasets not previously published but collected by authors are now provided in a table and in a figure in Supplementary Material Section 3.7 (showing G6PDd prevalence estimates). These data have been collected by co-authors and are published for the first time in this paper.

******

*If possible please reformat the citation style into PLOS Medicine's format (should be straight forward if using referencing software) - this should use callouts formatted as sequential numerals in square brackets (not superscript). Many thanks

We have modified all references and in-text citations to the PLOS Medicine format style using EndNote X8.

*Please clarify in the methods section whether the analytical approach reported in the paper corresponds to one laid out in a prospective protocol or analysis plan. Please state this (either way) early in the Methods section.

a) If a prospective analysis plan (from your funding proposal, IRB or other ethics committee submission, study protocol, or other planning document written before analyzing the data) was used in designing the study, please include the relevant prospectively written document with your revised manuscript as a Supporting Information file to be published alongside your study, and cite it in the Methods section. A legend for this file should be included at the end of your manuscript.

b) If no such document exists, please make sure that the Methods section transparently describes when analyses were planned, and when/why any data-driven changes to analyses took place.

c) In either case, changes in the analysis-- including those made in response to peer review comments-- should be identified as such in the Methods section of the paper, with rationale.

This study did not have a prospective analysis plan, although all research questions and study aims were formulated before study commencement and data acquisition. These aims were outlined in the form of a grant proposal/Concept Note prepared by our team. As a result, we have added the following state to the methods overview section in the main text to clarify this point: "The treatment scenarios to be modelled were specified in advance of epidemiological data acquisition" (lines 146-147).

*Please include in the Discussion section a more explicit (ie signposted) description of key limitations (and strengths) of the model, specifically noting any which might preclude drawing firm conclusions or make the key findings less secure. At the moment a number of issues of interpretation are discussed but none are explicitly described as limitations of the model per se.

We acknowledge that in our initial submission of the manuscript we overlooked the limitations of our work. After reflecting on how certain model assumptions and methodological approaches may not fully reflect real-world dynamics, we have identified several key limitations of our work. We have addressed these issues in the discussion (lines 503-516). We discuss limitations concerning our model not accounting for importations and relying on 2018 reported clinical cases to model community-level transmission. Such assumptions may not reflect the true transmission levels in 2021 when tafenoquine will be introduced including the potential impact of COVID-19 on health system disruptions.

Comments from the reviewers:

Reviewer #1: This is an important contribution on the long journey to malaria elimination in Latin America which will require tafenoquine. Most modelling papers are criticized for being overly optimistic which is not a feature of this manuscript; it clearly claims 'no silver bullet' against malaria. The importance of getting a pediatric formulation and dealing more with adult males (likely miners in the Brazilian context) is certainly made in the discussion. Although G6PD deficiency is clearly discussed, it is not belaboured which is good. One additional point could be considered is that all malaria programs require a working health system. Brazil's health system has been massively disrupted by COVID-19 and although better than Venezuela, that is not saying much in the American context. Models are based on what has happened up to a point, some mention that things can also go backwards when health systems are disrupted may need to be inserted.

I am neither a modeler nor a statistician so although I see no errors of fact or interpretation, would hope that the editor has brought in other reviewers with such skills to examine the numbers.

We thank the reviewer for their positive comments highlighting the public health relevance of our work and writing style. Indeed, while Brazil has generally a robust health system compared to other malaria endemic countries, we cannot ignore the fact that COVID-19 interrupted malaria case management. Given the timeframe set for the revision, we do not have the capacity to explore the impact of COVID-19 on malaria treatment in Brazil; however, we can draw from recently published work that has modelled the impact of COVID-19 on malaria transmission in endemic settings in Africa (Sherrard-Smith, E., Hogan, A.B., Hamlet, A. et al. The potential public health consequences of COVID-19 on malaria in Africa. Nat Med 26, 1411–1416 (2020) https://www.nature.com/articles/s41591-020-1025-y) . Sherrard-Smith et al.

have shown that disruptions in LLIN distribution, IRS, and malaria treatment would result in increasing *P. falciparum* transmission at differing magnitudes. If we assume disruptions to case management and treatment of malaria due to a largely unmitigated COVID-19 scenario in the Brazilian Amazon Region occurred this year, we would expect that a certain proportion of clinical cases may have gone untreated during the epidemic.

We agree that a statement should be made in the discussion regarding the possible effect of COVID-19 on access to malaria treatment (although LLIN and IRS occur, we assume no impact of these interventions since they are not routinely implemented in Brazil). As a result, we have added the following statements to the discussion of the limitations of our study: "Since the settings we modelled were calibrated to 2018 P. vivax incidence, we assumed constant transmission up to 2021 when tafenoquine is introduced. Therefore, we did not account for dynamic changes in incidence informed by historical trends. We also did not account for the possibility of potential health system distributions due to COVID-19. Drawing from recently published work that has modelled the impact of COVID-19 on malaria transmission in endemic settings in Africa [40], we would expect some level of disruption to malaria case management and thus an increase in incidence. A thorough assessment of the impact of COVID-19 on malaria transmission accounting for variability in seasonal trends of P. vivax as well as delayed introduction of the new treatment scheme is required." (lines 508-516). Indeed this would be a good future research question to explore with modelling.

Reviewer #2: I enjoyed reading this carefully written paper. The authors have modeled the impact of tafenoquine deployment on P. vivax malaria in Brazil. The underlying model is well thought through and the assumptions have been explained more than adequately. The main result - TQ will help reduce transmission but is not a silver bullet - is very important.
In the interests of reproducibility, could the authors make their code available?

We thank the reviewer for this question and agree that we overlooked sharing the link to the model code. We agree that public availability of the code for interests of reproducibility is important for validation of mathematical models. We have now added a reference to the website (https://github.com/MWhite-InstitutPasteur/Pvivax_TQ_IBM) in the Methods section (lines 158-159).

My only other comments concern the epistemic uncertainty around some of the modelling assumptions, and these are mainly cosmetic and should not impact the main results of the paper. All the comments pertain to the supplementary text, which is well worth reading!

G6PD deficiency: this is a nice analysis. It might be worth pointing out that the enzyme activity data (Fig S4) are all from countries with G6PD mutations that are thought to be more severe (i.e. lower activity in deficient red cells) than the A-202 mutation that is the major mutation in South America: Bangladesh has Orissa, Kalyan-Kerala and Mahidol; Cambodia has Viangchan; Indonesia has mostly Vanua Lava; Israel has Mediterranean. In particular Vanua Lava and Med are probably the most severe variants found at >1% allele frequency. Therefore your estimate of the number of heterozygous females below the 70% threshold is probably conservative (overestimate). It's also possibly worth mentioning that the data from Pal et al (African Americans) are directly applicable to Brazil as they will be most certainly A-202.

The reviewer has brought up an important point concerning the appropriateness of our modelling fitting for G6PD activity. While ideally we would have liked to fit the model with data from Brazil, we currently do not have access to such datasets. However, as the reviewer suggested, it is important to highlight how differences in the Bangladeshi, Cambodian, Indonesian, and Israeli populations could affect our results. In consequence, we have added the following statements:

In SI section 2.3:
"Since African Americans were included in the study and similar G6PDd genetic variants are observed between African Americans and Brazilians [10] (see section 2.4), we assume that the SD Biosensor G6PD test will have comparable classification between the two populations."

And in SI section 2.4 along with appropriate references:
"While the G6PDd prevalence model estimates are consistent with those reported by each study, G6PD mutations in these populations are considered more severe than the prevalent G6PD A(−) variant (202 G>A mutations) commonly found in South America.[11-13] The common variant forms in the datasets: Bangladesh (Orissa, Kalyan-Kerala and Mahidol); Cambodia (Viangchan); Indonesia (Vanua Lava); Israel (Mediterranean) tend to be more severe and as a result, may overestimate the G6PD activity in the Brazilian population.[14, 15] In particular, the prediction of heterozygous women under the 70% threshold in Brazil may be overestimated in comparison to the data used to fit the model. As a result, more women could in fact be eligible for tafenoquine treatment than predicted by the model. Overall, since this concerns a small proportion of the overall population, this will not have a major impact on our prediction of tafenoquine impact on transmission; however, further optimization of the model will be required using Brazilian data when it becomes available."

CYP2D6: I think that this section has the highest epistemic uncertainty and it would be useful to mention this. You rightly note "The association between CYP2D6 activity and the efficacy of 8-aminoquinolines against P. vivax hypnozoites is undoubtedly a complex one, with multiple sources of evidence combining to suggest a non-linear dose-response relationship between activity and efficacy."
I want to add a few comments on this complex picture.

1. Tafenoquine is not affected by CYP2D6: the oft quoted results from St Jean et al are unfortunately confounded. Their Fig 1 shows in the primaquine group a higher relapse frequency in intermediate metabolisers as compared to extensive metabolisers, but the opposite pattern in the tafenoquine group. But this result is meaningless because both relapse patterns and distribution of CYP2D6 is highly dependent on study country (DAG: relapse <- country -> CYP2D6)! I can't work out what the adjusted result would be without the raw data (only marginal distributions are given in the paper), but the effect could be the opposite (Simpson's paradox). Lacerda et al (NEJM) does not appear to adjust for country when analyzing the effect of CYP2D6 on primaquine recurrence.

In our opinion, the data from the St Jean *et al* is consistent with tafenoquine not being affected by low CYP2D6 metabolization, but we feel that this is a very low category of evidence, most notably due to the potential for confounding by study country as highlighted. Indeed, we also considered the impact of low CYP2D6 on tafenoquine efficacy in Scenario 8 and observed an 8.9% reduction in the median predicted impact (SI Section 4.7).

We have added the following statements the SI Section 3.6:
"While the surveyed data are certainly consistent with the efficacy of tafenoquine not being affected by low CYP2D6 metabolization, we do not consider this to be a very high standard of evidence due to the

potential for confounding. Therefore, we also implement sensitivity analyses where this assumption is varied."

2. Primaquine is affected by CYP2D6: the results from Kevin Baird and colleagues are very compelling. However, in our analysis of primaquine efficacy in vivax patients along the Thai-Myanmar border (Taylor et al, Nature Communications), we estimated a very low primaquine failure ratio (~3%). There are lots of intermediate metabolisers (AS=0.5 or 1) in this population. Unfortunately, we didn't put this in the paper, so you can't cite it, but for the record, out of 349 patients who were genotyped, 5 had an AS score = 0, 40 had AS=0.5; 90 had AS=1. There was no association between the AS score and estimated probability of primaquine failure. This may be due to a dose effect as these patients were given high dose primaquine. it may be due to the epidemiology or other factors we don't know. It also may be due to the scoring system - see next point.

We thank the reviewer for sharing their unpublished results which further underline the complexity of the relationship between primaquine and low CYP2D6 metabolisation. Since published data is scarce and as you previously mentioned, highly dependent on the country of study, patient factors, and primaquine dose, we found that the simplest approach was to apply a sensitivity analysis on the proportion of individuals who have some form of low CYP2D6 metabolisation that will lead to complete treatment failure.

We simulated 3 scenarios (S1,S9,S10) (see detailed results in SI Section 4.9): primaquine being completely ineffective in 4%, 8% and 20% of the population. The major assumptions here are that these prevalence estimates are due to how we define low CYP2D6 metabolisers. While this is an important factor in how we estimate primaquine efficacy and implement it into the model, the main objective of our study was to evaluate the impact of tafenoquine on transmission at a more macro-level. Despite overlooking some of the more finer details, we were still able to extrapolate interesting results from our sensitivity analyses: if some form of low CYP2D6 is expected to impact less than 10% of the population, we do not expect to see a high variability in impact (overlapping 95% uncertainty intervals across the median effect size for all simulated settings in S1 (8.1% low CYP2D6 prevalence) vs. S9 (4% low CYP2D6 prevalence); however, if low CYP2D6 prevalence is expected to be higher than 10% (S10; 20% low CYP2D6 prevalence) then we would expect to see more impact on treatment efficacy and as a result transmission dynamics at the population level.

Admittedly, our modelling does not go beyond this sensitivity analysis and takes a more simplistic approach to the problem. One possibility to overcome this is to develop a model to describe a non-linear dose-response relationship between low CYP2D6 activity and primaquine efficacy. Unfortunately, data to inform the model on quantitative low CYP2D6 metabolisation levels and on the particular primaquine regimen in Brazil (3.5 mg/kg total dose over 7 days) is lacking.

3. AS scores: this is a scoring system used by pretty much everyone in order to map genotype to phenotype. It's an incredibly useful because the phenotype test is very labor intensive. However, it has many issues. First there is large inter-individual variability for the same genotype (Chiba et al, Inter-individual variability of in vivo CYP2D6 activity in different genotypes 2012). Second, the mapping might not be very accurate for some of the genotypes. From the inventors of the system themselves (Andrea Gaedigk et al. Ten Years' Experience with the CYP2D6 Activity Score: A Perspective on Future Investigations to Improve Clinical Predictions for Precision Therapeutics. 2018):

"[..] the classification of alleles as increased, normal, decreased or no function is crude, and does not take into account substrate-dependent effects of an allelic variant toward various CYP2D6 substrates [7]. This challenge is perhaps best exemplified by CYP2D6*10, a decreased function allele, which has been associated with substantially reduced levels of activity. [..] the current assignment of a value of 0.5 to this allele classifies homozygous CYP2D6*10/*10 patients as NM in CPIC guidelines, which may not be appropriate for all drugs. A literature review and assessment performed in 2013 [8] did not produce sufficient evidence to recommend a reclassification of the CYP2D6*10 allele by assigning a lower value of 0.25 to better reflect the level of reduction of this allele at that time. However, a recent extensive literature review and assessment performed for the CYP2D6/tamoxifen gene/drug pair CPIC guideline produced strong evidence for a separate recommendation for
CYP2D6*10 containing genotypes [9]"

If I understand correctly, *10 constitutes about half the poor/intermediate metabolisers in Brazil? It's probably fair to say that there is considerable uncertainty as to the effect of CYP2D6 on the use of both tafenoquine or primaquine.

The reviewer has brought up another critical point concerning the uncertainty of the impact of low CYP2D6 and how it should be defined. Indeed, even the authors that developed the scoring system are in the process of reviewing their definitions. While previously stated, we accounted for some but not all the uncertainties around low CYP2D6 and varying prevalence. Overall, we expect that at the population level, lower CYP2D6 prevalence is expected to favor effective radical cure and lowering transmission while higher prevalence would result in the opposite. Going beyond such sensitivity analyses when modelling population-level impact may not significantly impact our macro-level results but indeed it would capture more potential heterogeneous dynamics. However, models at the individual level accounting for these complex metabolic factors and pharmacodynamics in the future will become very informative to macro-level modelling studies.

We have added our more simplified approach to addressing the complexities around low CYP2D6 metabolisation as a limitation in the Discussion with a reference to the 2018 paper by Gaedigk *et al*.

"Second, while treatment efficacy behaves on continuous scale, we treated drug efficacy and low CYP2D6 metabolisation as binary conditions and did not account for inter-individual heterogeneity.[39]" (lines 505-507)

Minor comments:
* Is one decimal point necessary in the abstract results?
* Line 40: for P. vivax
* Line 92: suggest changing this to "demonstrated non-inferiority with respect to low-dose primaquine to prevent relapse"
* Line 97: X-linked

Thank you for the minor comments and for pointing out some of the errors. We have integrated these suggestions/corrections via the following:

- Rounding to the nearest value in the abstract and removing the decimal (lines 31-32)
- Adding "for" in line 42
- Changing "In Phase 3 trials, it demonstrated comparable efficacy to prevent relapse with primaquine whilst requiring only the administration of a single dose" to "In Phase 3 trials, it

demonstrated non-inferiority with respect to low-dose primaquine to prevent relapse whilst requiring only the administration of a single dose" in lines 101-102

- We capitalized the "x" in "X-linked" in line 106

Reviewer #3:

It would be useful to discuss the selection of a Susceptible Infectious Susceptible model as opposed to a Susceptible Infectious Recovered one, say an extension of the McDonald or May-type, as often used in modelling Malaria

This is important since the long term dynamics of the different types of model are different

Indeed, we agree with the reviewer that the dynamics between an SIS and SIR model structure will be different, in particular in the long term. The current model we have implemented for our work has been previously described in detail by the senior authors of our current paper. The model developed by Dr. White is an extended Ross-Macdonald mathematical model of *P. falciparum* initially developed by Dr. Azra Ghani's unit at Imperial College that accounts for the relapse characteristics of *P. vivax*. While the model is neither an SIS or SIR per se, it does account for a Susceptible compartment, 3 Infected compartments, a Treated and Prophylaxis compartment that can be considered as a form of a "Recovered" compartment. We describe the model in more detail in Section 1 of the Supplementary Materials:

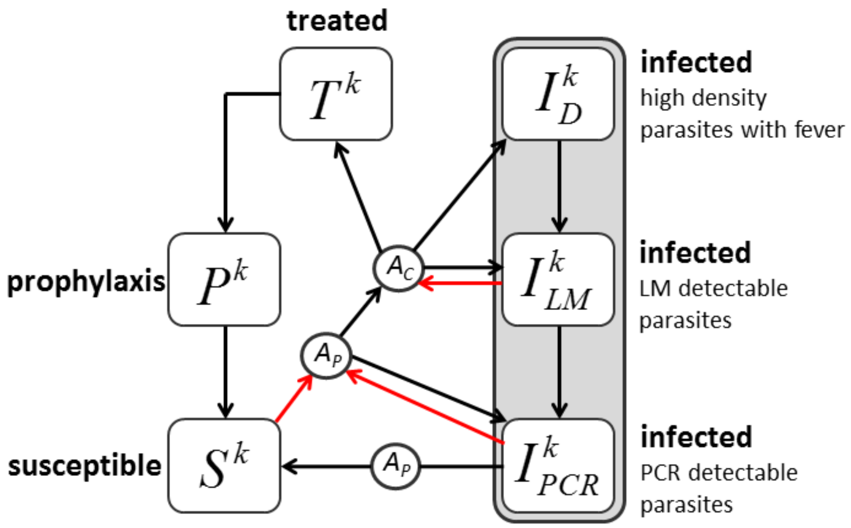

Figure S1: Compartmental representation of *P. vivax* transmission model in humans. Infected individuals can be in one of three compartments depending on whether blood-stage parasitaemia is detectable by PCR (IPCR), light microscopy (ILM) or has high density with accompanying fever (ID). A proportion of individuals that progress to a symptomatic episode of P. vivax will undergo treatment with a blood-stage drug (T) leading to clearance of blood-stage parasitaemia and a period of prophylactic protection (P) before returning to the susceptible state (S). The superscript k denotes the number of batches of relapse causing hypnozoites in the liver. Red arrows denote new blood-stage infections arising from either new mosquito bites or relapses. Each square denotes a compartment and the circles denote the dependence

of transition rates between compartments on levels of anti-parasite immunity (Ap) and levels of clinical immunity (Ac).

Also, given that malaria transmission is mostly local (at least within the 126 municipalities), did the authors consider a structured, say meta-population-type, model?

The reviewers make an interesting point suggesting the use of a meta-population model to explore *P. vivax* transmission dynamics as opposed to the independent municipality-level populations we modelled.

We have observed in our descriptive analyses of the Brazilian malaria case reports from 2003 to 2018 that between 6.7% and 17.6% of cases did not share the same municipality of notification, residence, and probable infection. In order to translate these proportions into potential mechanisms of inter-municipality transmission, more investigation is required in understanding the reporting behavior in the database. In particular, the reporting of municipality of probable infection is at the discretion of the health profession which can introduce bias. While we do think inter-municipality importations play an important role in very low transmission such as pre-elimination settings in Tocantins and Maranhão states (see the Figure 5C below from our publication (R Lana*, N Nekkab*, AM Siqueira, C Peterka, P Marchesini, M Lacerda, I Mueller, M White†, D Villela†, *The top 1% – quantifying the unequal distribution of malaria: the case of Brazil*) currently under review in Malaria Journal), we do not think importations have a significant role in moderate and high burden settings that are the main targets for tafenoquine case management.

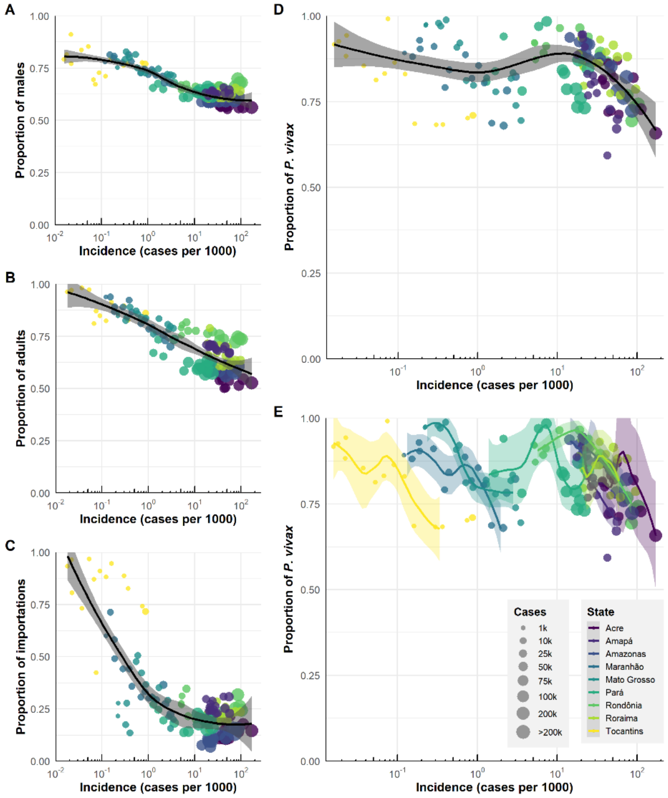

Figure 5. Characterizing the path to elimination. (A) The proportion of notified malaria cases in males increases in lower transmission settings. (B) The proportion of notified malaria cases in adults (>16 years) increases in lower transmission settings. (C) The proportion of notified malaria cases due to importation increases in lower transmission settings. (D) Across the Amazon Basin, the majority of notified malaria cases are due

to P. vivax. (E) When analysed on the state level, an increasing proportion of *P. vivax* cases is seen in lower malaria transmission settings. Each point represents one observation per state from 2004 to 2018. The loess curve is estimated using data from 2006 onwards and observations with at least 20 annual cases reported (A-D: for all points; E: by state).

Within the given timeframe for the revision of the manuscript, it would require a substantial investment of time and effort to develop and integrate a meta-population modelling approach for the endemic region of Brazil. Therefore, for our primary objective to explore the potential public health impact of introducing tafenoquine in Brazil, we believe our model is sufficiently detailed to account for the main drivers of changes in *P. vivax* transmission due to changes in case management practices. We are however open to the potential of developing a meta-population model in the future to answer questions related to case management practices and vector control intervention for a more localized community such as peri-urban Manaus where better data is available.

That would differentiate the stochastic and the deterministic models substantially, including their mean behaviour

The model development ensures approximately equal results between the deterministic and stochastic models for large population sizes (100,000 individuals per simulation). For small population sizes, we see the phenomenon of stochastic fade out (e.g. Figure 2G). The incorporation of importation would reduce the likelihood of stochastic fade out.

In relation to the model type, treatment efficacy and prevalence, are often assumed to be exchangeable, as opposed to identical, across regions/municipalities. This assumption is typically adopted in order to allow for similarity and heterogeneity simultaneously. Would that be an option in this paper?

Such an approach would incorporate Manaus-type heterogeneity in a natural manner

When good quality and reliable data was available at the municipal level (e.g. G6PDd prevalence, mean age, population size, incidence), we parameterized each setting accordingly. When data relied on international studies or assumptions (e.g. drug efficacy from clinical trials, low CYP2D6 impact, G6PD diagnostic performance), we assumed no difference across municipalities since there is no data to support such variations.

Please add the details of the algorithm used for fitting the Bayesian mixture model. In particular, what was the approach used for dealing with label switching?

A Markov Chain Monte Carlo algorithm with Metropolis Hastings updated was utilized. Minimally informative uniform priors were assumed. The priors also enforced a greater mean G6PD activity in normals than in deficients. This prior information prevents label switching.

Gaussian mixture model for males
q*dnorm(g6pd_males, mean=mu_def, sd=sig_def) + (1-q)*dnorm(g6pd_males, mean=mu_nor, sd=sig_nor)
Gaussian mixture model for females
(q^2)*dnorm(g6pd_females, mean=mu_def, sd=sig_def) + 2*q*(1-q)*dnorm(g6pd_females, mean=mu_het, sd=sig_het) + ($(1-q)^2$)*dnorm(g6pd_females, mean=mu_nor, sd=sig_nor)
Where we define q to be the prevalence of G6PD deficiency, based on the proportion of males with a deficient genotype, normal males (1 – q), homozygous deficient females q^2, heterozygous deficient

females 2q(1 – q), and homozygous normal females $(1 – q)^2$ based on the Hardy-Weinberg principles of genotype frequencies in the population at equilibrium. We define qnorm as the density normal distribution function, with a given series of data (the vector g6pd_males and g6pd_females as G6PD quantitative activity scores reported in the studies), and mean mu and standard deviation sig.

We addressed the potential issues of label switching by imposing priors on the mean values (mu_nor > mu_het > mu_def). These priors are informed by a large body of work that has assessed the range of quantitative G6PD activity scores across populations, often with genotyping data (Bancone, G., Gornsawun, G., Chu, C. S., Porn, P., Pal, S., Bansil, P., ... & Nosten, F. (2018). Validation of the quantitative point-of-care CareStart biosensor for assessment of G6PD activity in venous blood. PLoS One, 13(5), e0196716; Satyagraha, A. W., Sadhewa, A., Elvira, R., Elyazar, I., Feriandika, D., Antonjaya, U., ... & Harahap, A. R. (2016). Assessment of point-of-care diagnostics for G6PD deficiency in malaria endemic rural Eastern Indonesia. PLoS neglected tropical diseases, 10(2), e0004457).

Are the mosquito population dynamics essentially absent from the model?
Is there an implicit assumption that they remain the same or stationary over time? Please discuss

Our modelling framework accounts for heterogeneity and seasonality of exposure to mosquito bites; detailed mosquito bionomics and modelling of larval population densities. The feeding, resting and oviposition behaviour of adult female mosquitoes are described by a number of key entomological parameters including life expectancy, human blood index (HBI), proportion of day time biting, and indoor resting preferences. More detail can be found from the previous publication of the model: White, M.T., Walker, P., Karl, S. et al. Mathematical modelling of the impact of expanding levels of malaria control interventions on Plasmodium vivax. Nat Commun 9, 3300 (2018). https://doi.org/10.1038/s41467-018-05860-8

The 37% at risk assumption (supplement page 4) seems important but is informed by a future manuscript. Please give in the present paper a short description of the data and methods resulting in this number

For our 37% occupational risk assumption for males, we relied on data from a study conducted in Manaus:
[29] Monteiro W, Karl S, Kuehn A, Almeida A, White M, Vitor-Silva S, et al. Prevalence and Force of Plasmodium Vivax and Plasmodium Falciparum Blood Stage Infection and Associated Clinical Malaria Burden in the Brazilian Amazon. Malar J. 2020. doi: 10.21203/rs.3.rs-51282/v1.

The occupation and hazard ratios are reported in the study and were used to derive this estimate.

How extensive is the evidence, especially long term, on Tafenoquine? What is the best way to incorporate the associated uncertainty? Please discuss as this is a crucial component of the model and partly drives the results and conclusions.

The evidence for tafenoquine efficacy and safety has been demonstrated in the Phase 2 and Phase 3 trails in which we derive our parameter estimates and reference in detail in our Supplementary Information Section 3:

[6] Lacerda MVG, Llanos-Cuentas A, Krudsood S, Lon C, Saunders DL, Mohammed R, et al. Single-Dose Tafenoquine to Prevent Relapse of Plasmodium vivax Malaria. N Engl J Med. 2019;380(3):215-28. doi: 10.1056/NEJMoa1710775. PubMed PMID: 30650322; PubMed Central PMCID: PMCPMC6657226.
[45] Llanos-Cuentas A, Lacerda MVG, Hien TT, Vélez ID, Namaik-larp C, Chu CS, et al. Tafenoquine versus Primaquine to Prevent Relapse of Plasmodium vivax Malaria. New England Journal of Medicine. 2019;380(3):229-41. doi: 10.1056/NEJMoa1802537. PubMed PMID: 30650326.
[48] Llanos-Cuentas A, Lacerda MV, Rueangweerayut R, Krudsood S, Gupta SK, Kochar SK, et al. Tafenoquine plus chloroquine for the treatment and relapse prevention of Plasmodium vivax malaria (DETECTIVE): a multicentre, double-blind, randomised, phase 2b dose-selection study. The Lancet. 2014;383(9922):1049-58. doi: https://doi.org/10.1016/S0140-6736(13)62568-4.

There is strong evidence from the Phase 3 trial to support that tafenoquine demonstrated non-inferiority with respect to low-dose primaquine to prevent relapse. We assume hypothetical scenarios of higher efficacy estimates in modelled Scenarios 2, 4, and 7.
