# Supplementary material for: Estimated impact of tafenoquine for Plasmodium vivax control and elimination in Brazil: A modelling study
Source: PLoS Med. 2021 Apr 23;18(4):e1003535. doi: 10.1371/journal.pmed.1003535 (PMC8064585; doi:10.1371/journal.pmed.1003535)
Supplement: Attachment — Submitted filename: Response to revisions.pdf [file pmed.1003535.s003.pdf]

Revisions

Requests from the editors:

1. Title: Please update to: "Estimated impact of tafenoquine for Plasmodium vivax control and elimination in Brazil: A modelling study"

The manuscript title has been updated in the manuscript and in the supplementary file.

2. Please update your Competing Interests statement on the submission form to say: "The authors have declared that no competing interests exist."

We have updated our response accordingly.

3. Data Availability Statement: You state in our first question on the submission form that restrictions apply, then in the second question that all data are in the manuscript and its files. Please confirm which situation is applicable.
a. If the data are freely or publicly available, note this and state the location of the data: within the paper, in Supporting Information files, or in a public repository (include the DOI or accession number).
b. If the data are owned by a third party but freely available upon request, please note this and state the owner of the data set and contact information for data requests (web or email address). Note that a study author cannot be the contact person for the data.
c. If the data are not freely available, please describe briefly the ethical, legal, or contractual restriction that prevents you from sharing it. Please also include an appropriate contact (web or email address) for inquiries (again, this cannot be a study author).

Thank you for pointing out this issue. We have clarified our data availability statement as follows:

"The model code is publicly available online at https://github.com/MWhite-InstitutPasteur/Pvivax_TQ_IBM. Restrictions apply for SIVEP malaria case reporting data. SIVEP data is owned by the Ministry of Health of Brazil which can be attained by meeting data access criteria by contacting the Health System Informatics Department (DATASUS) at datasus@saude.gov.br. Demographic data is publicly available at https://www.ibge.gov.br/. All other relevant data for modeling calibrations are within the manuscript and its Supporting Information files."

4. Abstract:
a. Please add a sentence at the start to mention, eg. the total number of infections in 2018, and the proportion of vivax infections.

We added the following to the background section of the abstract:

"In 2018, over 237,000 cases of malaria were reported to the Brazilian health system of which 91.5% were due to *P. vivax*."

b. Line 25: Please specify the dates for data collected.

We added "in 2018" to specify the year we calibrated our transmission settings.

c. Line 31: Replace “expected” with “estimated”.

We replaced this term.

d. Please quantify your results with 95% CIs and p values.

In our study, we quantified uncertainty around the median or mean estimates by providing either the minimum and maximum range of values or 95% uncertainty intervals, which describe the 2.5% or 97.5% percentile of values from 100 iterations of model simulations. We did not apply any statistical tests, therefore there are no p-values provided. We updated our results to calculate the mean during the 2021 to 2030 period by averaging the median effective radical cure rate of 100 iterations of each setting per year. We also reported the 95% UI and have updated Table 4 accordingly.

We updated the text as follows:

“While achieving elimination in only few settings in Brazil, tafenoquine rollout in 2021 is estimated to improve the mean effective radical cure rate from 42% (95% UI [41%-44%]) to 62% (95% UI [54%-68%]) among clinical cases leading to a predicted 38% (95% UI [7%-99%]) reduction in transmission and over 214,000 cumulative averted cases between 2021 and 2025.”

e. Please give some additional details, e.g., the modelled year of 2021; relevant 5 year window; observed regimen adherence and assumed adherence to tafenoquine.

We added the year of tafenoquine introduction and the period of evaluation.

“While achieving elimination in only few settings in Brazil, tafenoquine rollout in 2021 is estimated to improve the mean effective radical cure rate from 42% (95% UI [41%-44%]) to 62% (95% UI [54%-68%]) among clinical cases leading to a predicted 38% (95% UI [7%-99%]) reduction in transmission and over 214,000 cumulative averted cases between 2021 and 2025.”

f. Line 37: Please clarify that this is “case importations”?

We modified the text as follows:

“..the role of imported cases from outside the transmission setting, …”

g. Conclusions: Begin with: “In our modelling study, we predicted that…”

We have updated this sentence with this text:

“In our modelling study, we predicted that concurrent rollout of G6PDd diagnostics…”

h. Line 42 (and later 518): Please replace “silver bullet” with a different term, to avoid terms all readers may not be familiar with.

We have made the following changes:

In the abstract:

“While tafenoquine alone may not be sufficient for *P. vivax* elimination…”

In the discussion:
“…one should not expect tafenoquine alone to lead to *P. vivax* elimination.”

5. Author Summary:

a. Overall, we would recommend shortening your Author Summary by 1-2 bullet points per section. Please also adjust the tone to be more suitable to a lay reader, and avoid too much technical language, eg. “haemolytic events”, “G6PDd” (unless you define it first), “phenotypic activity”, “CYP2D6 metabolisation”, “peri-domestic”.

b. Line 51 (and throughout, eg line 100): Please remove brand names.

c. Line 69: Please say “first, to our knowledge” or similar.

d. Line 77: Please delete: “Even in a setting such as Brazil with strong pre-existing case management with primaquine, we would not expect tafenoquine to be the silver bullet that will lead to P. vivax elimination.”

We have accounted for all of these points and have updated the author summary as follows:

“Why was the study done?

- Radical cure with tafenoquine plus chloroquine has been recently approved to treat P. vivax malaria; however, the impact of improving individual-level effective radical cure by overcoming non-compliance with primaquine with this single dose regimen on population-level transmission is unknown.
- Depending on age, pregnancy, and lactating status, glucose-6-phosphate dehydrogenase (G6PD) prevalence along with drug metabolisation, treatment eligibility and effective radical cure rates may vary across different populations with variable transmission.

What did the researchers do and find?

- With mathematical modelling, we accounted for these complex dynamics to consider the non-linear dynamics of 8-aminoquinoline treatment on the burden of P. vivax malaria across a range of settings and for various implementation strategies.
- To our knowledge, our work is the first to show how rolling out tafenoquine in populations will improve case management of P. vivax, reduce transmission and prevent a substantial number of cases even in settings with high rates of effective case management with primaquine. However, tafenoquine alone will not lead to P. vivax elimination.

What do the findings mean?

- Accounting for the factors that lead to a variable degree of impact, our results can guide countries considering introducing tafenoquine with G6PD deficiency testing as part of tailored intervention strategies adapted to their local context.
- Overall, tafenoquine will have several public health benefits; however, it should be considered as an additional tool along with other interventions to reach elimination goals in the proposed timeframe.”

6. Please move your citations to before punctuation and remove spaces within the citation, eg: “…strategy tackling P. vivax malaria [7,8].”

We have modified all in-text citations to this format.

7. Methods:

a. Line 161: Please give exact date ranges for the case reports used.

We added:

"…from January to December 2018"

b. Please state that separate ethical approval was not required for your study.

We added:

"Ethical approval was not required for our study."

8. The terms gender and sex are not interchangeable (as discussed in https://urldefense.com/v3/__http://www.who.int/gender/whatisgender/en/__;!!JFdNOqOXpB6UZW0!_huxVlYaoIKNV0OsVgj9G9lRVH44oyCE2lRiu5vGfc2VJoU2hG7YdqjVGE8ynL-8yjtLTOg$); please use the appropriate term.

We replaced all previous references to gender to sex as it is the more appropriate term.

9. Please provide a URL or DOI for reference 23, and spell out the full author organisation name.

This change has been made to ref 23.

10. Please check that journal short names are presented consistently in your reference list, eg. should be "PLoS Med".

We have updated all references and checked that all references are consistent with one another including journal names and abbreviations.
